# Synergizing Covalent‐Ionic Tri‐Site Coordination in P2‐type Na_0.67_Fe_0.33_Mn_0.67_O_2_ Induces Suppressed Layer‐Slipping and Stablized Lattice Oxygen for Sodium‐Ion Batteries at Wide Potential

**DOI:** 10.1002/advs.76648

**Published:** 2026-07-21

**Authors:** Haitao Xue, Xudong Shi, Zhiyue Lian, Wenxiu He, Genqiang Zhang, Yongqiang Zhang

**Affiliations:** ^1^ School of Chemistry and Chemical Engineering Inner Mongolia University of Science & Technology Baotou Inner Mongolia China; ^2^ Hefei National Research Center for Physical Sciences At the Microscale Department of Materials Science and Engineering University of Science and Technology of China Hefei Anhui China

**Keywords:** cathode, covalent‐ionic balancing, energy storage, layered oxide, sodium‐ion battery

## Abstract

Improving the stability of the oxygen framework and suppressing irreversible interlayer slip remain long‐standing challenges for Fe/Mn‐based layered oxide cathodes. Herein, a trans‐sublattice three‐site co‐substitution strategy is proposed and implemented in Na_0.67_Fe_0.08_Cu_0.25_Mn_0.62_Mg_0.05_O_1.95_F_0.05_ (NFCMMOF) to precisely regulate the covalent‐ionic bonding balance within both cationic and anionic sublattices. This rational lattice design simultaneously mitigates the detrimental P2‐O2 phase transition and Jahn‐Teller distortion, constructs highly robust TM‐O/F bonds, markedly reinforces the overall oxygen framework, and establishes a highly reversible Fe^3+^/Fe^4+^‐dominated charge compensation mechanism. In situ XRD, in situ EIS‐DRT, and comprehensive DFT calculations collectively reveal that the synergistic three‐site regulation significantly enhances layered structural stability, expands the O‐Na‐O slab spacing, lowers Na^+^ migration barriers, and enables the concurrent optimization of charge transport and ion diffusion kinetics. Consequently, NFCMMOF delivers a high reversible capacity of 162.9 mAh·g^−1^ at 0.1 C and retains 85.1% of its capacity after 1000 cycles at 10 C, together with outstanding moisture tolerance. Moreover, the assembled full cell achieves high energy densities of 281.5 and 160.8 Wh·kg^−1^ at power densities of 70.4 and 2260.7 W·kg^−1^, respectively, demonstrating a broadly applicable lattice‐engineering paradigm for fast, stable, and practical Na^+^ storage.

## Graphical abstract

Trans‐sublattice Cu/Mg/F three‐site coordination simultaneously stabilizes the oxygen framework and suppresses irreversible P2‐O2 phase transition and Jahn‐Teller distortion in Fe/Mn‐based layered cathodes. The optimized NFCMMOF enables highly reversible Fe3+/Fe4+ redox chemistry, accelerated Na+ transport, and robust interfacial kinetics, delivering exceptional rate capability, ultralong cycling stability, and high‐energy‐density sodium‐ion full cells.

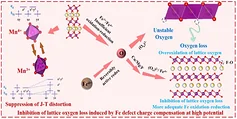

## 1 Introduction

Lithium‐ion batteries (LIBs), the cornerstone of modern electrification, were honored with the 2019 Nobel Prize in Chemistry for their profound impact on energy storage technologies [1, 2, 3, 4, 5, 6]. Nevertheless, the scarcity of lithium resources, supply instabilities, and rapidly increasing cross‐industry demand have driven up production costs, motivating the exploration of alternative, low‐cost energy storage systems [7, 8, 9, 10, 11]. Owing to the natural abundance of sodium and its comparable electrochemical behavior to lithium, sodium‐ion batteries (SIBs) have emerged as a highly promising substitute for LIBs [12, 13, 14, 15]. Despite these advantages, achieving high‐performance SIBs remains challenging, particularly in developing cathode materials that deliver high energy density, excellent rate capability, and robust long‐term stability. Consequently, the design and optimization of advanced sodium‐based cathode materials have attracted substantial research attention in recent years [16, 17, 18, 19, 20, 21].

Sodium‐ion battery (SIB) cathode materials are generally divided into three main categories: layered transition‐metal oxides (LTMOs), polyanionic compounds, and Prussian blue analogues. Among these, LTMOs have garnered particular attention due to their high theoretical capacity, well‐defined Na^+^ diffusion channels, environmental benignity, and ease of large‐scale synthesis. Depending on the Na^+^ coordination environment and the stacking sequence of oxygen layers, LTMOs can be further classified into two representative structural types: P2 and O3 phases [22, 23, 24, 25, 26]. Among the layered oxide family, P2‐type materials possess a more open Na^+^ diffusion framework, where Na^+^ occupies the trigonal prismatic (e/f) sites between TMO_2_ slabs. Such an architecture offers superior structural robustness and fast ionic transport, endowing these materials with outstanding rate capability and cycling stability. However, P2‐type cathodes still face severe challenges at high voltages (>4.0 V), including irreversible P2‐O2 phase transitions accompanied by significant lattice shrinkage, limited Fe^3+^/Fe^4+^ redox participation, and oxygen‐related charge compensation processes that trigger lattice oxygen release and oxygen‐sublattice distortion [27, 28]. In addition, the strong sensitivity of P2‐type oxides to moisture and air further hinders their practical implementation [29, 30]. Consequently, advanced lattice and interfacial engineering strategies are essential to construct P2 cathodes featuring glide‐resistant frameworks, highly reversible transition‐metal‐centered redox activity, and robust oxygen sublattices, together with moisture‐tolerant or hydrophobic surfaces. Such integrated structural and interfacial optimizations can synergistically enhance energy density, rate capability, and long‐term durability.

To address the aforementioned challenges, various modification strategies have been developed, including elemental doping, surface coating, and morphology engineering. Among these, elemental doping (e.g., Co^3+^, Fe^3+^, Cu^2+^, Mg^2+^, Zn^2+^, Al^3+^, and F^−^) has proven particularly effective in enhancing phase‐transition reversibility and optimizing Na^+^ diffusion pathways. Previous studies revealed that substituting Fe^3+^ with Cu^2+^ can stabilize the P2 framework and raise the operating potential, thereby improving the overall energy density [31]. Meanwhile, Mg^2+^, serving as an electrochemically inert stabilizer, suppresses structural degradation and facilitates more reversible phase evolution [32]. However, single‐element doping alone is inadequate to sustain superior electrochemical performance above 4.0 V, where intricate coupled redox reactions and phase transitions dominate the electrochemical process [33]. Therefore, recent efforts have focused on co‐doping strategies that integrate active and inert multi‐ions, enabling concurrent regulation of crystal and electronic structures. Such synergistic design aims to simultaneously achieve high‐rate capability, environmental stability, and prolonged cycling durability [34]. Accordingly, Wang et al. implemented Mg/F co‐doping in NNMO, which effectively suppressed lattice‐oxygen loss and mitigated the irreversible P2‐O2 phase transition, thereby enhancing the cycling stability of the cathode [35]. In parallel, Mg and Cu co‐substitutions effectively improve ambient stability, smooth voltage profiles, and reinforce the structural robustness of layered frameworks under high‐voltage operation [36, 37]. However, most reported layered transition‐metal oxides (LTMOs) still suffer from pronounced air and moisture sensitivity, limited capacity retention, and rapid performance degradation under high‐voltage operation. Moreover, elucidating the synergistic interactions between anionic and cationic sublattices, as well as the associated structure‐performance relationships, is crucial for guiding the rational design of advanced layered oxide cathodes [38].

In this work, a Cu/Mg/F co‐doping strategy is proposed to realize cross‐sublattice three‐site substitution in P2‐type Na_0.67_Fe_0.33_Mn_0.67_O_2_ (NFMO), enabling the precise co‐regulation of the covalent‐ionic bonding balance within the layered framework. This rational lattice‐engineering approach effectively suppresses irreversible interlayer sliding and structural phase transitions under high‐voltage operation. The introduced TM─O/F bonding configuration stabilizes the oxygen framework, while the charge‐compensation mechanism is shifted toward a highly reversible Fe^3+^/Fe^4+^‐dominated redox pathway. Benefiting from this cross‐sublattice synergistic regulation, the optimized Na_0.67_Fe_0.08_Cu_0.25_Mn_0.62_Mg_0.05_O_1.95_F_0.05_ (NFCMMOF) cathode exhibits outstanding electrochemical performance, delivering high reversible capacities of 162.9 and 88.9 mAh·g^−1^ at 0.1 and 10 C, respectively, together with excellent cycling stability. Notably, after 200, 500, and 1000 cycles at 1, 5, and 10 C, the capacity retentions remain as high as 90.62%, 91.14%, and 85.14%, respectively. By integrating in situ XRD, in situ EIS‐DRT, and comprehensive DFT calculations, it is systematically demonstrated that NFCMMOF maintains a highly stable oxygen‐framework layered structure, expands the O‐Na‐O interlayer spacing, reduces the Na^+^ migration energy barrier, and achieves synergistic optimization of electron transport and ion diffusion kinetics. Importantly, when coupled with a commercial hard carbon anode, the NFCMMOF full cell delivers a stable capacity of 75.9 mAh·g^−1^ at 5 C with a capacity retention of 80.1% after 400 cycles, and achieves high energy densities of 281.5 and 160.8 Wh·kg^−1^ at power densities of 70.4 and 2260.7 W·kg^−1^, respectively. Beyond the specific material system investigated, this work establishes a broadly applicable cross‐sublattice co‐doping paradigm for simultaneously stabilizing lattice frameworks, modulating charge‐compensation chemistry, and accelerating ion‐transport kinetics, thereby providing a feasible design blueprint for next‐generation high‐rate and long‐life sodium‐ion battery cathodes.

## 2 Results

Na_0.67_Fe_0.08_Cu_0.25_Mn_0.62_Mg_0.05_O_1.95_F_0.05_(NFCMMOF) and the corresponding control samples were synthesized via a sol‐gel route followed by high‐temperature calcination. The precursor metal salts were dissolved according to the stoichiometric ratios, with citric acid serving as the complexing agent. Detailed synthesis procedures are provided in the Supporting Information. To elucidate the structural evolution induced by Cu, Mg, and F co‐doping, X‐ray diffraction (XRD) analyses were performed on all samples. As presented in Figure S1, all diffraction patterns display sharp and well‐defined peaks indexed to the typical P2‐type layered oxide structure (space group P63/mmc, JCPDS No. 27–0751), confirming that multi‐site doping does not alter the parent phase. Notably, the diffraction peaks of NFCMMOF shift slightly toward lower angles compared with NFMO, suggesting an expansion of lattice parameters. To further quantify the lattice variations caused by Cu/Mg/F co‐doping, Rietveld refinements were conducted for NFMO, NFCMO, NFCMMO, and NFCMMOF (Figure 1a,b and Figure S2 and Tables S1–S4). Compared with NFMO (a = 2.91572 Å, c = 11.34567 Å; Figure S3), NFCMO exhibits a reduced c‐axis length, likely due to the strong electron‐withdrawing nature of Cu^2+^, which lowers the Mn^3+^/Mn^4+^ ratio. Upon the sequential introduction of Mg and F, the c‐axis parameter progressively increases, reaching its maximum in NFCMMOF. As illustrated in the refined structural model (Figure 1c,d), Cu^2+^, Mg^2+^, and F^−^ occupy the Fe (2a), Mn (2a), and O (4f) sites, respectively. The O‐Na‐O interlayer spacing expands from 3.53985 Å in NFMO to 3.56365 Å in NFCMMOF. This expansion is primarily attributed to the reduced negative charge density of the transition‐metal–oxygen layers upon Cu/Mg/F substitution, which weakens the Coulombic attraction between the Na layers and TM‐(O/F) slabs. The resulting local structural relaxation facilitates Na^+^ diffusion and aligns well with previously reported observations for optimized P2‐type layered oxides [35].

**FIGURE 1:**
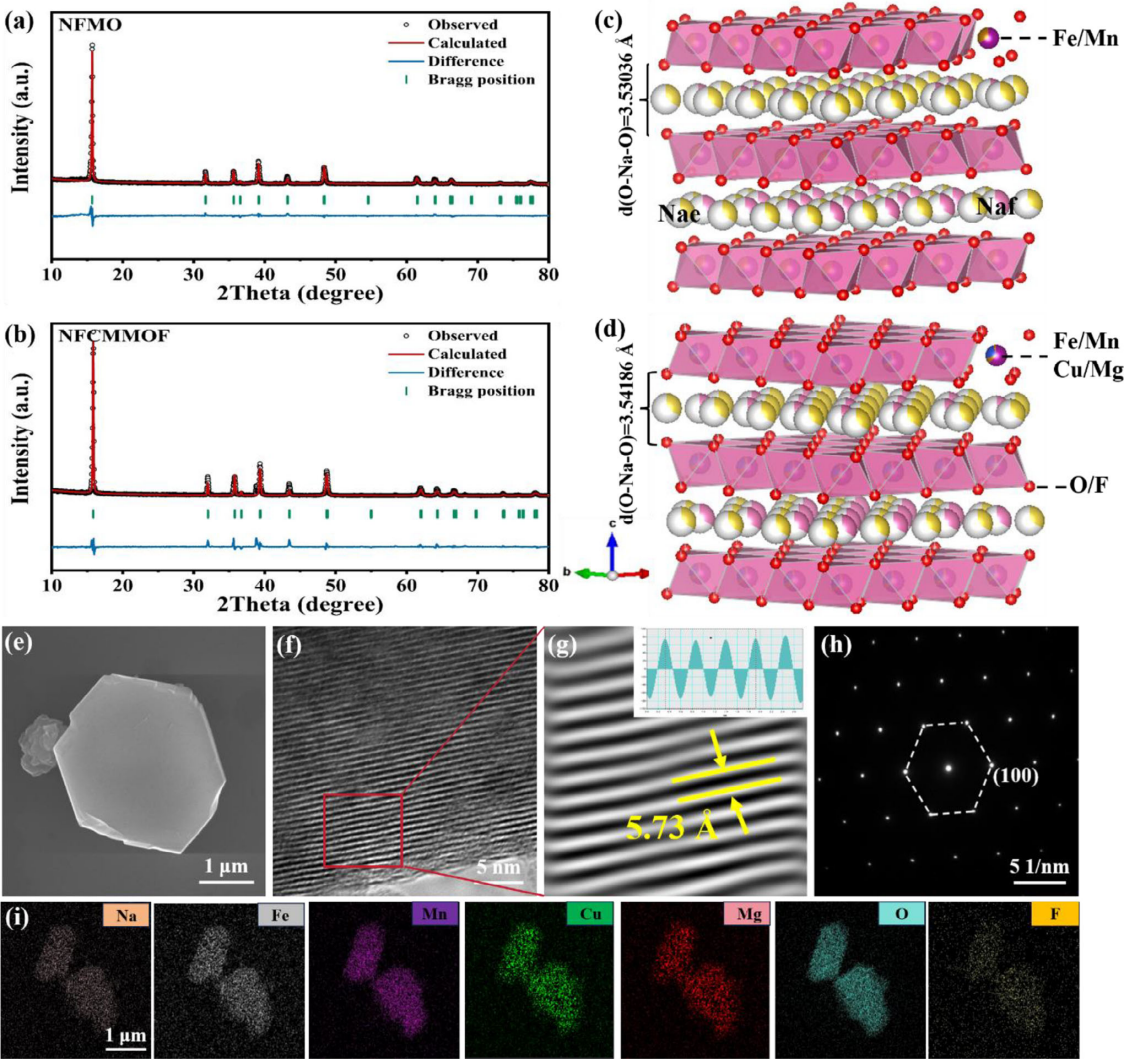
Morphological and Structural properties. Rietveld refinement of XRD patterns of (a) NFMO and (b) NFCMMOF. Crystal structure diagrams of (c) NFMO and (d) NFCMMOF. (e) SEM image of NFCMMOF. (f–g) HRTEM image of NFCMMOF. (h) SAED image of NFCMMOF. (i) EDS image of NFCMMOF.

Scanning electron microscopy (SEM) images show that all samples exhibit clear micron‐sized flake crystals with obvious layered characteristics (Figure 1e and Figure S4). It is worth noting that the crystal shape of NFCMMOF shows a typical hexagonal crystal morphology with a smooth surface, and the crystallinity is better than that of NFMO, NFCMO, and NFCMMO. In addition, the particle distribution of NFCMMOF is relatively loose, while the remaining materials show significant particle agglomeration (Figure S5), which will improve the wetting of the electrolyte to the electrode material and generate a more uniform current distribution during the cycle, further enhancing the electrolyte penetration [39]. High resolution transmission electron microscopy (HRTEM) images (Figure 1f,g and Figures S6–S8) clearly reveals the parallel lattice fringes in NFCMMOF, and the lattice spacing is about 5.73 Å, while the lattice spacing of NFMO, NFCMO and NFCMMO is smaller than that of NFCMMOF, which are 5.67, 5.61 and 5.70 Å, respectively, which are well corresponding to the (002) crystal plane. These observations are consistent with the XRD results, confirming the increase in the interlayer spacing of NFCMMOF. Therefore, the TEM image further proves the excellent layered periodicity, indicating that the co‐doping does not destroy the ordered stacking of the layered structure. Selected area electron diffraction (SAED, Figure 1h) shows clear diffraction spots pointing to the (100) crystal plane, showing high crystallinity and ordered layered stacking consistent with P2. Energy dispersive spectroscopy (EDS) spectra (Figure 1i and Figures S9–S11) showed that Na, Fe, Mn, Cu, Mg, O, and F elements were evenly distributed throughout the sample, and no area of element agglomeration or enrichment was detected. This confirms that the uniform introduction of Cu/Mg/F is beneficial to maintain a uniform reaction interface during the cycle, thereby preventing local stress accumulation and poor side reactions. In addition, inductively coupled plasma optical emission spectroscopy (ICP‐OES) analysis verified that the elemental composition of NFCMMOF was well matched with the designed molar ratio (Tables S5–S8).

To investigate the synergistic effects of Cu, Mg, and F in the three‐site substitution system, an NFCMMOF structural model was constructed (Figure S12a,b), in which Cu^2+^, Mg^2+^, and F^−^ occupy the Fe‐2a, Mn‐2a, and O‐4f sites, respectively, followed by comprehensive density functional theory (DFT) calculations. The site selection of Cu^2+^/Mg^2+^/F^−^ is based on crystal‐chemical matching and energetic stability criteria (see below for detailed discussion). Bader charge analysis (Table S9), combined with differential charge density maps (Figure 2a, b), demonstrates that Cu/Mg/F co‐doping enables coupled regulation of charge distribution and bonding characteristics within the TM‐(O/F) lattice, with the regulatory effect extending throughout the entire framework. In NFMO, charge enrichment and depletion are predominantly confined to isolated TM─O bonds, indicative of a highly localized charge‐compensation mode dominated by ionic interactions, and no effective cross‐layer charge coupling or transport pathway exists between the Na layers and the TM─O framework. Such localized and ionicity‐dominated charge distribution readily amplifies local polarization and promotes lattice oxygen loss under high‐voltage conditions, thereby accelerating lattice distortion and phase‐transition instability. In contrast, NFCMMOF exhibits significantly enhanced continuity of charge enrichment/depletion iso‐surfaces across the TM‐(O/F) framework, progressively forming a more interconnected through‐layer charge‐transport network. This delocalized charge redistribution reflects a transition toward a more cooperative bonding regime with enhanced covalent character in the oxygen framework, thereby rebalancing the covalent‐ionic bonding nature of TM‐(O/F) interactions. As a result, Na‐layer polarization is effectively alleviated, and a more robust electronic‐structure foundation is established for stabilizing both the layered lattice and the oxygen skeleton, while facilitating more reversible charge compensation. Consistent with these observations, the Bader charges of TM sites in NFCMMOF differ markedly from those in NFMO, indicating an enhanced participation of the TM sublattice in charge regulation upon co‐doping. Meanwhile, compared with unsubstituted oxygen sites (O1), the F‐substituted oxygen sites (O2) exhibit the most pronounced charge redistribution, demonstrating that F incorporation is not a simple iso‐valent substitution but instead induces site‐specific reconstruction of the local bonding environment, further optimizing the covalent‐ionic bonding balance and the chemical stability of TM‐(O/F) bonds.

**FIGURE 2:**
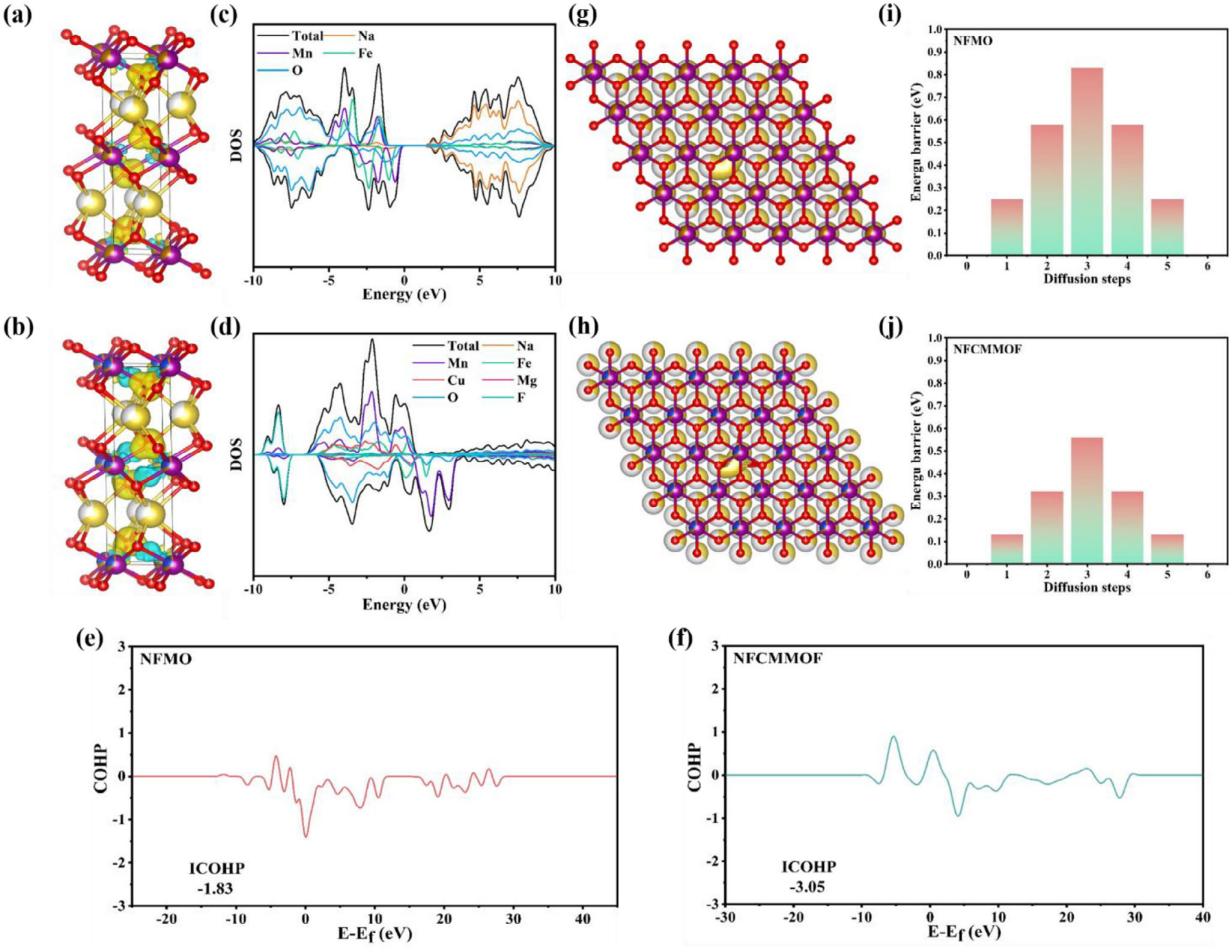
DFT calculation. (a) Differential charge density simulation image of NFMO. (b) Differential charge density simulation image of NFCMMOF after Cu/Mg replaces the TM 2a site and F replaces the O 4f site. (c) DOS density of states of NFMO. (d) DOS density diagram of NFCMMOF. COHP calculation for the change of TM‐O bond strength: (e) NFMO and (f) NFCMMOF. (g) NFMO cathode diffusion path diagram. (h) NFCMMOF cathode diffusion path diagram. (i) The change of diffusion energy barrier in NFMO. (j) The change of diffusion energy barrier in NFCMMOF.

Density of states (DOS) and crystal orbital Hamilton population (COHP) analyses further elucidate how Cu/Mg/F co‐doping enables the synergistic optimization of charge transport and TM‐O framework stability. As shown in Figure 2c, the valence‐band maximum of NFMO is almost entirely dominated by O‐2p states, whereas the contributions from Mn/Fe‐3d orbitals near the Fermi level are relatively limited. This electronic‐structure feature indicates that, under deep desodiation or high states of charge, lattice oxygen is more susceptible to excessive loss, thereby undermining the stability of the oxygen framework. By contrast, NFCMMOF exhibits a markedly enhanced density of states near the Fermi level (Figure 2d), which is primarily derived from Fe‐3d states with a certain degree of hybridization with Cu‐3d orbitals. The increased electronic‐state density near the Fermi level suggests improved electronic conductivity after multi‐element co‐doping, which in turn facilitates charge‐transport capability. Meanwhile, the O‐2p band center shifts toward lower energies, while the F‐2p states remain deeply located and electrochemically inactive, collectively indicating stabilization of the anionic framework. Within this reconstructed electronic environment, electrochemically inert Mg^2+^ does not directly contribute states near the Fermi level but instead modulates TM‐O interactions via an inductive effect, thereby fine‐tuning the covalent‐ionic bonding balance and the associated charge‐compensation chemistry. To quantitatively evaluate the impact of the above bonding‐balance reconstruction on structural stability, COHP analysis was further performed to characterize the TM─O bonding characteristics (Figure 2e,f). The integrated COHP (ICOHP) values reveal that the absolute ICOHP of NFCMMOF is significantly larger than that of NFMO (−3.05 vs −1.83), clearly indicating that co‐doping substantially strengthens the TM─O bonds and enhances their covalent character. In addition, the energy‐resolved COHP profiles show that NFMO still exhibits pronounced antibonding‐state contributions near the Fermi level, suggesting that its TM─O bonds are more susceptible to electronic perturbations during electrochemical cycling. By contrast, NFCMMOF is dominated by bonding states near the Fermi level, while the antibonding states are effectively shifted away from the operational energy window, thereby endowing the TM─O framework with intrinsically higher stability during Na^+^ deintercalation. Combined with the significantly reduced Na^+^ migration energy barriers obtained from DFT calculations, these results collectively demonstrate that Cu/Mg/F co‐doping not only enhances charge transport and structural stability, but also provides additional kinetic advantages for Na^+^ transport by synergistically optimizing the covalent‐ionic bonding balance (Figure 2g–j).

To clarify the synergistic effect of Cu/Mg/F co‐doping, the electrochemical performance of half‐cells was evaluated within a voltage window of 2.0–4.2 V using Na metal as the counter electrode. Figure 3a and Figure S13 present the galvanostatic charge‐discharge (GCD) profiles of NFMO, NFCMO, NFMMO, NFMOF, NFCMMO, NFMMOF, and NFCMMOF cathodes over the first three cycles at 0.1 C (1 C = 200 mA·g^−1^). For NFMO, an evident high‐voltage plateau appears during the initial charging process, corresponding to the irreversible P2‐O2 phase transition, in good agreement with previous reports [40, 41]. After three cycles, the discharge capacity decreases markedly, and the voltage plateau becomes significantly shortened, indicating pronounced irreversible structural distortion. Upon the introduction of Cu, Mg, and F dopants, the GCD profiles undergo substantial evolution. In NFMMO, a small voltage plateau around 3.4 V suggests that although Mg doping slightly improves cycling stability, the electrochemical behavior remains influenced by Na^+^/vacancy ordering. This ordering effect is partially alleviated by further F incorporation in NFMMOF; however, residual ordering features are still observed. In contrast, Cu‐doped NFCMO and NFCMMO exhibit significantly improved cycling stability. Nevertheless, the pronounced contraction of interlayer spacing induced by Cu^2+^ significantly hinders Na^+^ diffusion, and subsequent Mg doping does not lead to a notable increase in capacity. These results indicate that single‐element or binary doping strategies still possess inherent limitations and are insufficient to comprehensively optimize the electrochemical performance of the cathode materials. Notably, the Cu/Mg/F co‐doped NFCMMOF displays GCD profiles without an obvious voltage plateau above 4.0 V and exhibits nearly overlapping charge‐discharge curves, indicating that irreversible lattice distortion is effectively suppressed. This conclusion is further supported by subsequent in situ XRD measurements and post‐cycling morphological characterization. In addition, the smooth and highly symmetric voltage profiles of NFCMMOF signify highly reversible Na^+^ (de)intercalation behavior and accelerated redox kinetics.

**FIGURE 3:**
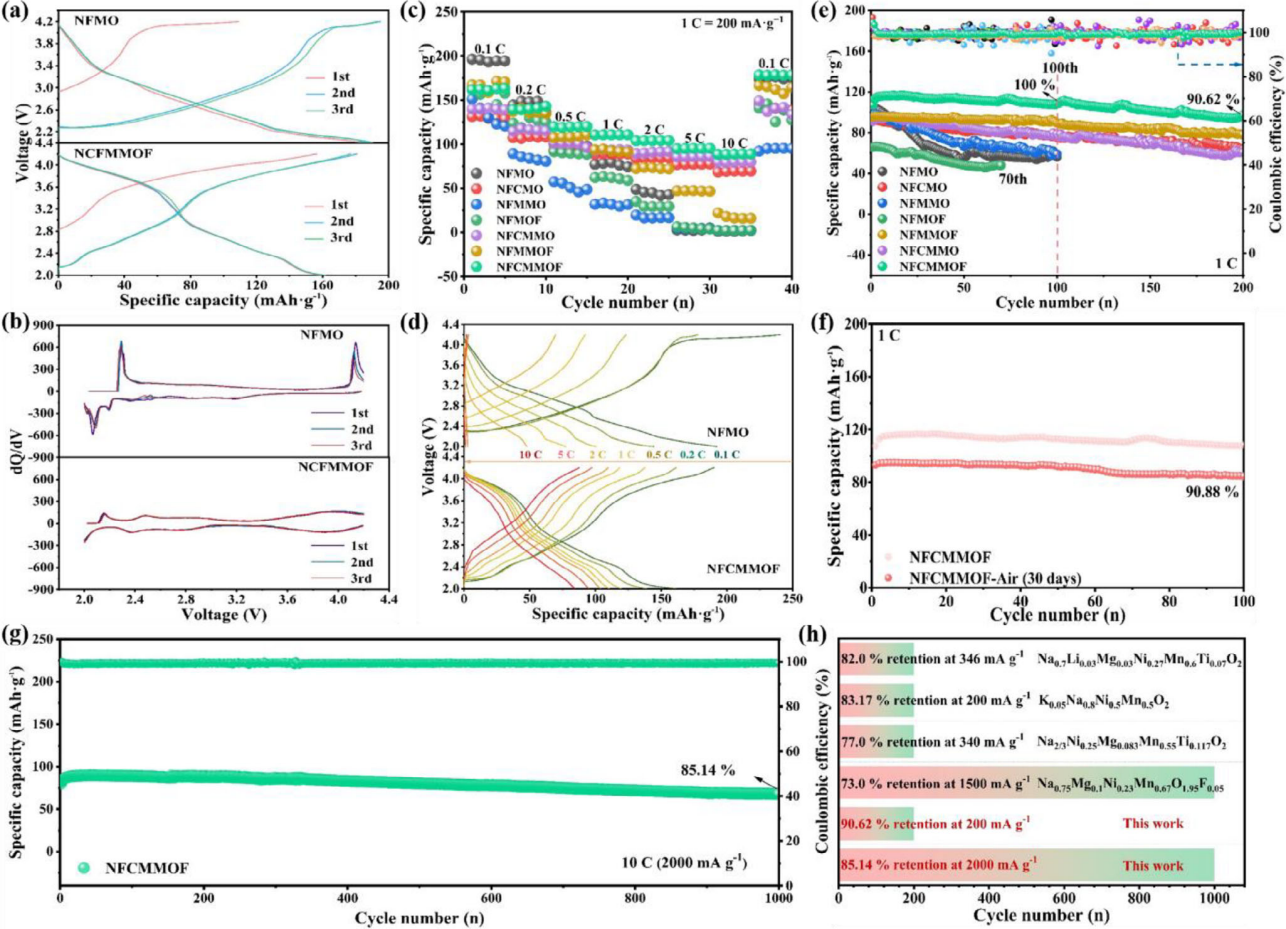
Electrochemical performance of NFCMMOF on a half‐cell configuration with Na metal. (a) Comparison of GCD curves of NFMO and NFCMMOF at 0.1 C. (b) Differential capacity curves of NFMO and NFCMMOF at 0.1 C. (c) Rate performance test comparison. (d) Comparison of GCD curves of NFMO and NFCMMOF at different magnifications. (e) Long cycle test performance at 1 C rate. (f) 1 C rate cycling test of NFCMMOF after 30 days of exposure to moist air. (g) Long cycle test performance at 10 C rate. (h) The cycle performance of NFCMMOF was compared with other reported electrode materials.

The differential capacity (dQ/dV) curves shown in Figure 3b and Figure S14 further substantiate the above analysis. NFMO exhibits a sharp oxidation peak at high potential, accompanied by pronounced peak attenuation upon cycling, indicative of severe irreversible structural changes under high‐voltage operation. With the successive introduction of different dopant ions, a clear evolution of redox behavior can be observed across the NFMO, NFCMO, NFCMMO, and NFCMMOF cathodes. Among these samples, NFCMMOF displays a broader, more symmetric, and highly overlapping dQ/dV profile over the entire voltage range, reflecting more reversible redox reactions and enhanced structural stability. Such well‐retained and symmetric redox features indicate reduced polarization and improved reaction reversibility during repeated Na^+^ insertion/extraction.

As shown in Figure 3c,d and Figure S15, the rate performance of NFMO and its co‐doped counterparts was evaluated over a wide current‐density range (0.1 C‐10 C‐0.1 C). Among all samples, NFCMMOF exhibits outstanding rate capability. Specifically, the discharge capacities of NFCMMOF at 0.1, 0.2, 0.5, 1, 2, 5, and 10 C are 162.9, 143.0, 120.1, 110.7, 104.1, 95.3, and 88.9 mAh·g^−1^, respectively. Notably, when the current density is reverted to 0.1 C, the discharge capacity is fully recovered to 178.4 mAh·g^−1^, even exceeding its initial value, corresponding to a capacity retention of over 100%. This remarkable behavior demonstrates the exceptional reversibility and structural robustness of NFCMMOF under high‐rate operation. In contrast, NFMO, NFCMO, NFMMO, NFMOF, NFCMMO, and NFMMOF exhibit considerably inferior rate performance. In particular, pristine NFMO shows extremely poor rate capability, delivering only 2.1 mAh·g^−1^ at 10 C. Moreover, upon returning to 0.1 C, only 89.6% of its initial capacity is recovered, indicative of severe kinetic limitations and pronounced irreversible degradation. The pronounced capacity recovery of NFCMMOF observed during the rate‐performance test can be attributed to the widely reported gradual electrochemical activation process of layered oxide cathodes. During cycling with progressively increasing current densities, electrode wetting and electrolyte penetration become more complete, while a stable cathode‐electrolyte interphase (CEI) is gradually established, leading to a continuous reduction in interfacial polarization. Consequently, Na^+^ transport accessibility and the utilization of reversible capacity are significantly improved. EIS measurements conducted before and after the rate‐cycling process (Figure S16) further corroborate this interpretation. For NFMO, the charge‐transfer resistance (R_ct_) increases from 527.8 to 591.4 Ω after rate cycling, indicating aggravated polarization and deteriorated interfacial kinetics. In contrast, the R_ct_ of NFCMMOF decreases from 213.7 to 176.2 Ω after rate cycling, demonstrating reduced polarization and markedly enhanced charge‐transfer kinetics, which highlights the pronounced synergistic advantages enabled by Cu/Mg/F co‐doping.

To evaluate the long‐term cycling stability of the cathode materials, NFMO, NFCMO, NFMMO, NFMOF, NFCMMO, NFMMOF, and NFCMMOF were systematically tested at a current density of 1 C, as shown in Figure 3e. At 1 C, NFCMMOF delivers an initial discharge capacity of 107.6 mAh·g^−1^ and maintains nearly 100% capacity retention after 100 cycles. Even after 200 cycles, a reversible capacity of 97.5 mAh·g^−1^ is retained, corresponding to a capacity retention of 90.62%. This excellent cycling stability significantly outperforms that of NFMO and the single or dual‐doped cathodes, including NFMO (53.97% after 100 cycles), NFCMO (69.19% after 200 cycles), NFMMO (62.5% after 100 cycles), NFMOF (72.91% after 70 cycles), NFCMMO (81.97% after 200 cycles), and NFMMOF (66.74% after 200 cycles), highlighting the clear advantage of the triple‐doping strategy. Furthermore, high‐rate cycling tests conducted at 5 C (Figure S17) reveal that NFCMMOF still retains 91.14% of its initial capacity (95.9 mAh·g^−1^) after 500 cycles. In sharp contrast, NFMO delivers only 9.1 mAh·g^−1^ under the same conditions.

To evaluate the moisture resistance of the cathode, NFMO and NFCMMOF were exposed to a humid air environment for 30 days. As evidenced by the FTIR and XRD results (Figures S18 and S19), the NFMO sample exhibited new diffraction peaks at approximately 13°, whose intensity exceeded that of the (002) reflection, along with an additional peak near 25°. Combined with the FTIR spectra, as well as previous reports [42, 43], these new features can be attributed to the formation of hydrated NaHCO_3_ degradation products. In contrast, NFCMMOF showed no sign of such secondary phase formation, confirming its superior structural stability under humid conditions. Mechanistically, the substitution of F^−^ for O^2−^ reduces surface Lewis basicity and lowers the O‐2p band center, thereby weakening the interlayer electrostatic attraction and suppressing moisture‐induced intercalation. Mg^2+^ incorporation at the 2a site effectively mitigates Jahn‐Teller distortion of Mn^3+^ and releases local strain, narrowing water diffusion channels and enhancing the lattice rigidity. Meanwhile, Cu^2+^ strengthens TM‐O covalency and improves electronic conduction, buffering interfacial polarization and impedance growth under humid exposure. Even after 100 cycles at 1 C (Figure 3f), NFCMMOF retained 91.72% of its initial capacity, whereas NFMO exhibited a pronounced capacity decay under identical conditions (Figure S20). This demonstrates its strong potential for stabilizing sodium‐ion battery operation under practical operating environments.

The NFCMMOF electrode exhibits excellent high‐rate cycling stability. During prolonged cycling at 10 C (2 A·g^−1^), it delivers an initial discharge capacity of 80.1 mAh·g^−1^ and retains 68.2 mAh·g^−1^ after 1000 cycles, corresponding to a high‐capacity retention of 85.14% (Figure 3g). As summarized in Figure 3h and Table S10, compared with recently reported P2‐type layered transition‐metal oxides (LTMOs), the Fe/Mn‐based LTMO developed via the Cu/Mg/F cross‐sublattice co‐doping strategy achieves a balanced regulation of covalent and ionic bonding characteristics. This regulation effectively stabilizes the layered oxygen framework while simultaneously enhancing charge‐transfer and Na^+^ transport kinetics. Benefiting from its Co/Ni‐free and low‐cost composition, NFCMMOF delivers an attractive combination of excellent high‐rate capability and prolonged cycling life, underscoring its strong potential for practical sodium‐ion battery applications.

To elucidate the structural evolution induced by the synergistic optimization of the Cu/Mg/F co‐doping strategy, in situ X‐ray diffraction (XRD) measurements were carried out during the charge‐discharge processes of NFMO and NFCMMOF within a voltage window of 2.0–4.2 V. As shown in Figure 4a,b, both NFMO and NFCMMOF exhibit evident diffraction‐peak shifts upon Na^+^ extraction: the (002) and (004) reflections gradually shift toward lower angles, while the (100) reflection shifts toward higher angles. These peak shifts indicate lattice expansion along the c‐axis and contraction along the a(b)‐axis during the desodiation process. In the high‐voltage region, a new diffraction peak appears at approximately 20° in NFMO (Figure 4a,c), which can be attributed to the formation of the O2 phase, thereby confirming the occurrence of a pronounced P2‐O2 phase transition, consistent with previous reports [44]. Owing to the substantial lattice mismatch between the P2 and O2 phases, the structure fails to fully revert to the original P2 phase during the subsequent discharge process (Figure S21), resulting in irreversible structural evolution. Moreover, as shown in Figure 4c and Figure S22a, an obvious splitting of the (004) reflection is observed at low voltages, which can be ascribed to interlayer sliding associated with the P2‐O2 transition as well as Mn^3+^‐induced Jahn‐Teller distortion. These structural instabilities collectively lead to severe lattice degradation and poor electrochemical reversibility. In sharp contrast, NFCMMOF maintains a single P2 phase throughout the entire charge‐discharge process without the emergence of any impurity phase (Figure 4b,d), indicating that the Cu/Mg/F co‐doping strategy effectively suppresses the P2‐O2 phase transition. This enhanced phase stability is mainly attributed to the retention of Na^+^ within the O‐Na‐O slabs, which reduces electrostatic repulsion between adjacent oxygen layers and effectively suppresses interlayer sliding of the TMO_2_ slabs. Consequently, the (004) diffraction peak of NFCMMOF remains sharp and symmetric throughout cycling, reflecting highly reversible structural evolution (Figure S22b). Furthermore, Rietveld refinement results (Figure 4e,f) reveal that NFMO undergoes a lattice‐volume change of approximately 3.41%, while the volume evolution of the corresponding O2 phase in the high‐voltage region is presented in Figure S23. In contrast, the total lattice‐volume change of NFCMMOF is limited to only 0.37%, demonstrating a significantly suppressed volume fluctuation. These results highlight the crucial role of the co‐doping strategy in enhancing structural stability and electrochemical performance.

**FIGURE 4:**
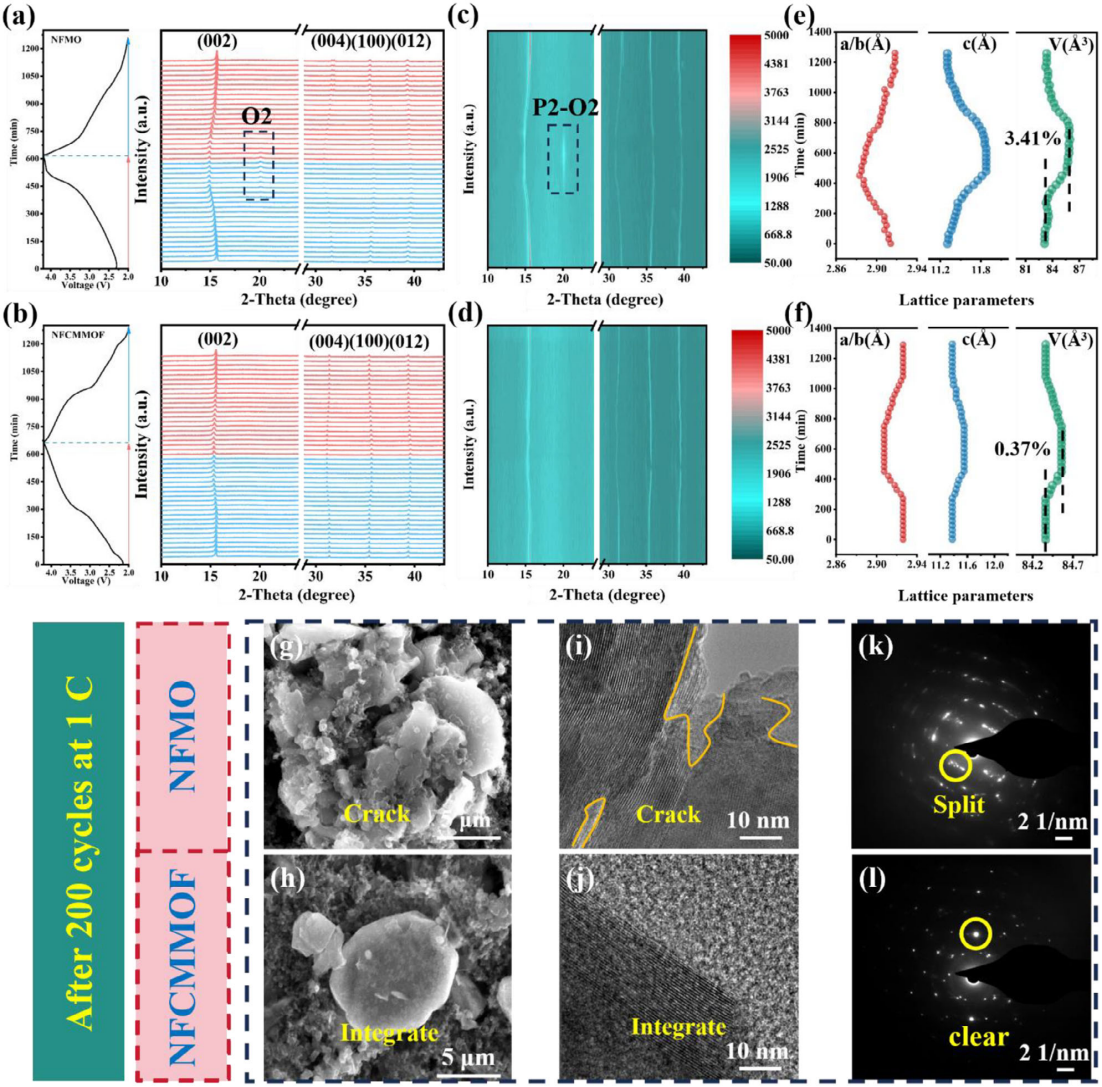
In situ XRD analysis and crystal morphology detection after cycling. In situ XRD patterns of (a) NFMO and (b) NFCMMOF. The intensity contour maps corresponding to (c) NFMO and (d) NFCMMOF. The variation curves of unit cell parameters corresponding to (e) NFMO and (f) NFCMMOF. Morphology analysis image after cycle: SEM image of (g) NFMO and (h) NFCMMOF. TEM image of (i) NFMO and (j) NFCMMOF. SAED image of (k) NFMO and (l) NFCMMOF.

To further clarify the effect of co‐doping on crystal integrity during prolonged cycling, ex situ XRD, SEM, and TEM analyses were performed on NFMO and NFCMMOF cathodes after 200 cycles at a rate of 1 C. As shown in Figure S24, the XRD pattern of NFMO after cycling exhibits pronounced structural degradation, with several characteristic diffraction peaks, such as the (100) reflection, almost completely disappearing. This observation reveals severe lattice collapse and structural disorder induced by long‐term electrochemical cycling. In contrast, the XRD pattern of NFCMMOF retains well‐defined diffraction features even after 200 cycles at 1 C, indicating excellent structural durability. SEM observations further show that NFMO undergoes pronounced particle cracking and fragmentation (Figure 4g), which can be attributed to repeated interlayer sliding and Jahn‐Teller distortion, leading to the accumulation of mechanical stress during cycling. In stark contrast, NFCMMOF preserves intact particle morphology with a compact and smooth surface (Figure 4h), demonstrating superior mechanical integrity and strong resistance to fatigue‐induced pulverization. TEM analyses shown in Figure 4i,j further corroborate these findings. NFMO exhibits fractured lattice planes (highlighted in yellow), and the corresponding selected‐area electron diffraction (SAED) pattern displays evident diffraction‐spot splitting associated with the (100) plane (Figure 4k), indicating severe phase disorder and lattice distortion. By contrast, NFCMMOF maintains clear and continuous lattice fringes along with sharp and well‐ordered SAED diffraction spots (Figure 4l), confirming its excellent crystallinity and structural integrity even after prolonged cycling. Overall, the structural stability and crystal integrity of NFCMMOF are significantly enhanced, demonstrating the effectiveness of Cu/Mg/F co‐doping in optimizing TM‐O bonding characteristics. This synergistic regulation alleviates internal strain accumulation, suppresses interlayer sliding, and collectively enhances the mechanical robustness and long‐term cycling durability of NFCMMOF.

X‐ray photoelectron spectroscopy (XPS) was employed to investigate the valence‐state evolution of NFMO and NFCMMOF electrodes during electrochemical cycling. As shown in Figure S25a, the survey spectra of NFMO, NFCMO, NFCMMO, and NFCMMOF clearly confirm the presence of all constituent elements, indicating their successful incorporation into the layered structure. For pristine NFMO, the Mn 2p spectrum (Figure S25b) exhibits characteristic peaks at 641.81 and 653.37 eV, corresponding to Mn 2p3/2 and Mn 2p1/2, respectively. Upon Cu substitution, the Mn 2p peaks of NFCMO shift slightly toward higher binding energies (641.85/653.41 eV), suggesting partial oxidation of Mn. A further positive shift is observed for the dual‐doped NFCMMO (641.91/653.49 eV), indicating a higher Mn oxidation state than that in NFCMO. Notably, in the Cu/Mg/F co‐doped NFCMMOF, the Mn 2p peaks shift more prominently to 641.97 and 653.87 eV, reflecting a substantial decrease in local electron density and a further increase in the Mn oxidation state. Quantitative fitting reveals that the Mn^4+^ fraction in NFCMMOF reaches 78.9 at%, markedly higher than that in NFMO (43.8 at%), indicating effective suppression of Jahn‐Teller distortion [45, 46]. Analysis of the Mn 3s spectra (Figure S26) further supports this conclusion. Based on the empirical relationship AOS = 8.956−1.126ΔE_s_, where ΔE_s_ represents the multiplet splitting, NFCMMOF exhibits a ΔE_s_ value of 4.53 eV, corresponding to an average oxidation state of 3.86, confirming the dominance of Mn^4+^ species [35]. As shown in Figure S25c,d, NFMO undergoes pronounced Mn^3+^/Mn^4+^ valence fluctuations during cycling, with Mn^3+^ remaining a major component even after discharge, which is detrimental to structural reversibility. In contrast, NFCMMOF maintains a consistently high Mn^4+^ fraction throughout the entire electrochemical process, with only minor variation during charge and discharge, indicative of a more stable and reversible Mn redox behavior. The Fe 2p spectra (Figure S25e,f) further reveal pronounced differences between the two materials. The Fe 2p spectrum exhibits characteristic peaks at 710.8 eV (Fe 2p3/2) and 724.7 eV (Fe 2p1/2), along with a satellite feature at 718.6 eV, confirming the presence of Fe^3+^. Simultaneously, the signals observed at 714.6 eV (Fe 2p3/2) and 728.3 eV (Fe 2p1/2) are indicative of Fe^4+^. In the pristine state, Fe predominantly exists as Fe^3+^ in both electrodes. Upon charging to 4.0 and 4.2 V, the fraction of Fe^4+^ in NFCMMOF is significantly higher than that in NFMO, reaching 70.1 at% at full charge, whereas NFMO exhibits only 28.6 at%. During the subsequent discharge process, Fe^4+^ in NFCMMOF can be effectively reduced back to Fe^3+^, indicating a highly reversible Fe^3+^/Fe^4+^ redox activity. In addition, the Cu 2p spectra (Figure S27) reveal a reversible Cu^2+^/Cu^3+^ redox couple, which plays an auxiliary role in the overall electrochemical activity. The Cu 2p spectrum displays characteristic peaks at 933.7 eV (Cu 2p3/2) and 953.6 eV (Cu 2p1/2), along with additional peaks at 936.2 eV (Cu 2p3/2) and 955.9 eV (Cu 2p1/2), as well as a satellite feature at 944.6 eV, confirming the presence of Cu^2+^ and Cu^3+^. Collectively, the Cu/Mg/F co‐doped framework establishes a clear redox hierarchy, in which Fe serves as the primary and highly reversible redox center, Cu provides auxiliary redox activity, and Mn is predominantly stabilized in a high‐valence state, thereby jointly promoting enhanced electrochemical reversibility and improved structural robustness.

To evaluate the evolution of oxygen‐related species during electrochemical cycling, O 1s XPS spectra were collected for NFMO and NFCMMOF electrodes at different states of charge (Figure S25g,h). The component located at 529.5 eV can be assigned to lattice oxygen (O^2−^), whereas the features in the range of 530.5–530.8 eV are commonly associated with oxidized or non‐stoichiometric oxygen species [47, 48]. Upon charging to 4.0 V, both electrodes exhibit an enhanced contribution in the 530.8 eV region, indicating the activation of oxygen‐related states at high voltage. Notably, NFMO shows a pronounced attenuation of the lattice O^2−^ signal, whereas NFCMMOF retains a substantially stronger lattice‐oxygen component, suggesting improved stability of the oxygen framework [49]. At full charge (4.2 V), the lattice‐oxygen signal in NFMO is nearly depleted, indicative of severe destabilization of lattice oxygen, while NFCMMOF still preserves a considerable fraction of lattice O^2−^. During the subsequent discharge process, even at 2.0 V, the lattice‐oxygen component in NFCMMOF remains largely recoverable, in sharp contrast to the irreversible oxygen loss observed in NFMO. Overall, although oxygen‐related states are activated at high voltage, NFCMMOF effectively stabilizes lattice oxygen and maintains the integrity of the oxygen framework (Figure S28), consistent with the results from in situ XRD and DFT calculations.

To explicitly elucidate the kinetic advantages enabled by Cu/Mg/F co‐doping, cyclic voltammetry (CV), in situ electrochemical impedance spectroscopy (EIS), and galvanostatic intermittent titration technique (GITT) measurements were performed to comparatively investigate the charge‐transfer behavior and Na^+^ diffusion dynamics of NFMO and its doped counterparts. As shown in Figure 5a and Figure S29, NFCMMOF exhibits well‐defined, highly stable current responses over a wide range of scan rates, whereas the pristine NFMO suffers from a rapid attenuation of current intensity at elevated scan rates, indicative of intrinsically sluggish kinetics and poor rate adaptability. The CV curves recorded at a low scan rate of 0.1 mV·s^−1^ (Figure S30) further reveal a clear evolution of electrochemical behavior across the NFMO‐NFCMO‐NFCMMO‐NFCMMOF series, highlighting the unique advantages of the ternary co‐doping strategy. NFMO displays steep and unstable anodic peaks above 4.0 V, accompanied by broadened and hysteretic cathodic peaks and low‐potential shoulder features. These characteristics reflect strong Na^+^/vacancy ordering, sluggish Na^+^ diffusion, and the activation of P2‐O2 slab gliding and oxygen‐related irreversible reactions under high‐voltage operation. Single Cu doping partially suppresses the abnormally high‐voltage oxidation peak in NFCMO and smooths the overall CV profile, indicating the activation of the Fe^3+^/Fe^4+^ redox couple and partial mitigation of phase‐transition behavior. However, residual Na^+^ ordering signatures and polarization remain evident, suggesting that Cu substitution alone is insufficient to fully stabilize the layered framework. With additional Mg incorporation, NFCMMO exhibits more symmetric and overlapping redox peaks, reflecting improved reversibility of the Mn^3+^/Mn^4+^ couple and reduced polarization at high potentials. This improvement is primarily associated with the suppression of Jahn‐Teller distortion and microstrain by Mg^2+^, which broadens Na^+^ diffusion channels but does not completely eliminate high‐voltage instabilities. In sharp contrast, the ternary co‐doped NFCMMOF demonstrates the most ideal electrochemical behavior, characterized by highly overlapped and smooth CV curves across the entire voltage window, minimal peak separation, and the complete disappearance of abnormal high‐voltage features. This superior performance indicates that Cu/Mg/F co‐doping simultaneously regulates cationic redox activity, lattice distortion, and anionic stability, thereby decoupling Na^+^ diffusion from structural instability. As a result, NFCMMOF establishes a highly reversible and kinetically robust electrochemical reaction pathway that cannot be achieved by single or double doping strategies alone.

**FIGURE 5:**
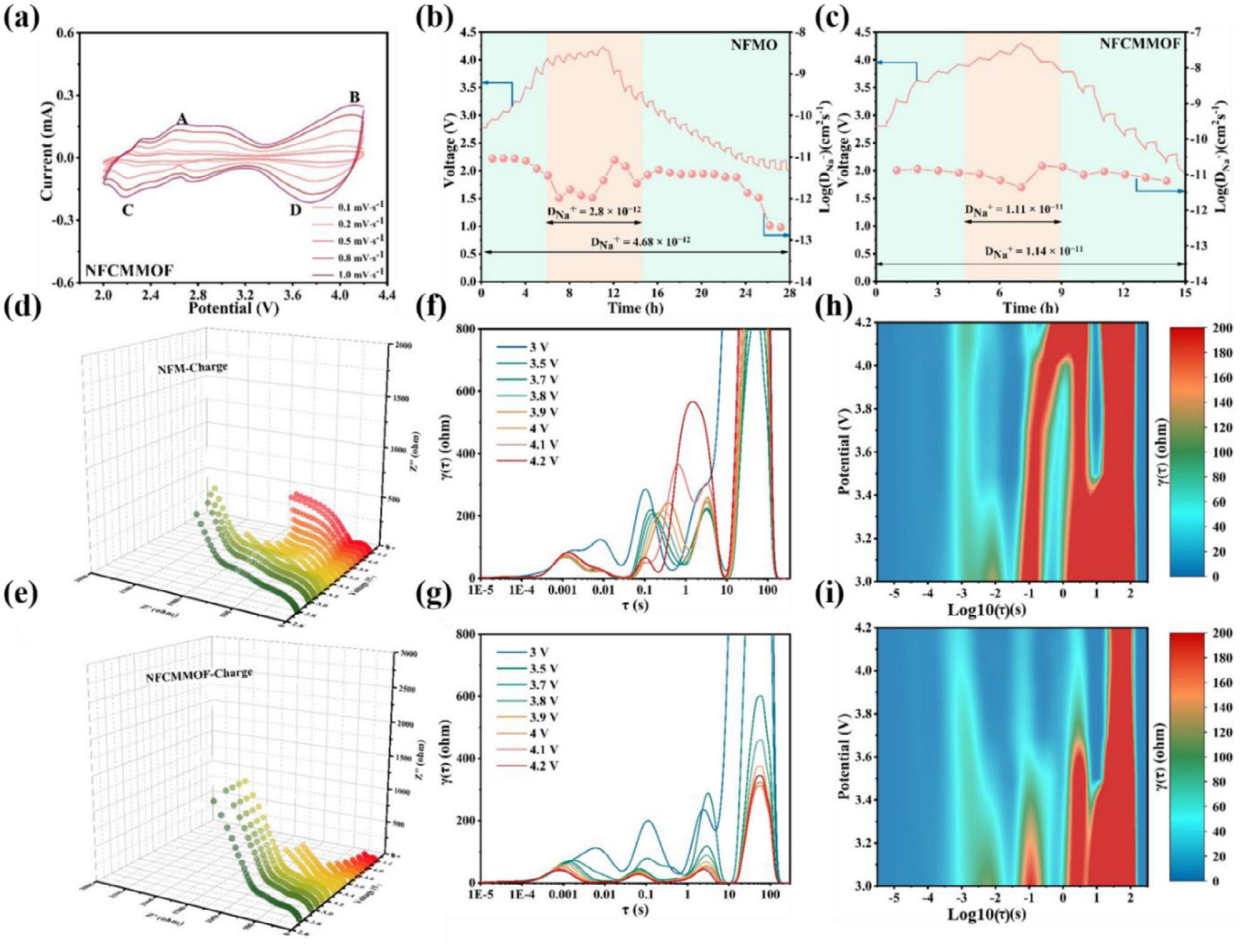
Changes in ion and charge transport dynamics of NFCMMOF. (a) CV curves of NFCMMOF at different scan rates. (b) GITT curve and D_Na_^+^ change of NFMO. (c) GITT curve and D_Na_^+^ change of NFCMMOF. In situ EIS 3D image of charging process: (d) NFMO; (e) NFCMMOF. The corresponding relaxation time distribution (DRT) curve during charging is measured by potential‐based in situ electrochemical impedance spectroscopy (In situ EIS): (f) NFMO; (g) NFCMMOF. DRT results contour map: (h) NFMO; (i) NFCMMOF.

To quantitatively assess the pseudocapacitive contribution, the CV data at different scan rates were analyzed using the Randles‐Sevcik equation [50]. According to the relationship i = av^b^, where i is the peak current and v is the scan rate, the fitted b values for NFMO and NFCMMOF are 0.80 and 0.96, respectively (Figure S31). The b value of NFCMMOF approaches unity, indicating that its electrochemical kinetics are predominantly governed by pseudocapacitive‐controlled processes rather than solid‐state diffusion. Furthermore, the total current response was deconvoluted using the equation i = k_1_v + k_2_v^1/2^, where k_1_v represents the capacitive‐controlled contribution and k_2_v^1/2^ corresponds to the diffusion‐controlled contribution. As shown in Figures S32 and S33, when the scan rate increases from 0.1 to 1.0 mV·s^−1^, the pseudocapacitive contribution of NFCMMOF increases from 86% to 97%, which is consistently higher than that of pristine NFMO across the entire scan‐rate range. The increase in pseudocapacitive contribution observed in Figure S32 highlights the predominance of surface‐controlled charge storage in NFCMMOF, which leads to highly reversible redox kinetics. This behavior enables faster charge/discharge cycles and effectively decouples the electrochemical reaction rate from the bulk Na^+^ diffusion limitations. These kinetic features are consistent with the excellent rate capability and long‐term cycling stability of NFCMMOF.

During the initial charge‐discharge cycle, the Na^+^ diffusion coefficients (D_Na_^+^) were systematically evaluated over different voltage regions based on GITT analysis. Notably, NFCMMOF exhibits a peak D_Na_^+^ of 1.11 × 10^−11^ cm^2^·s^−1^ in the high‐voltage region above 4.0 V, which is nearly four times higher than that of NFMO (2.97 × 10^−12^ cm^2^·s^−1^) (Figure 5b,c). This pronounced enhancement highlights the intrinsic advantage of Cu/Mg/F co‐doping in promoting fast Na^+^ transport under high states of charge, where layered cathodes are typically most vulnerable to kinetic degradation. In sharp contrast, NFMO shows a pronounced decrease in D_Na_^+^ and obvious polarization in the same voltage range (Figure 5b), which can be attributed to interlayer sliding and structural disruption induced by the P2‐O2 phase transition at high voltages. Such structural instability significantly impedes Na^+^ migration by narrowing diffusion pathways and increasing local energy barriers. Over the entire charge–discharge process, NFCMMOF maintains a relatively stable Na^+^ diffusion behavior across the full voltage window, with an average D_Na_^+^ of 1.14 × 10^−11^ cm^2^·s^−1^, whereas the average value for NFMO is only 5.36 × 10^−12^ cm^2^·s^−1^. These results are in excellent agreement with the first‐principles‐calculated diffusion barriers and collectively confirm that Cu/Mg/F co‐doping effectively optimizes the lattice framework, suppresses detrimental structural distortions, and stabilizes lattice oxygen, thereby enabling consistently enhanced Na^+^ transport kinetics throughout electrochemical cycling.

To further evaluate the charge‐transfer kinetics, in situ electrochemical impedance spectroscopy (In situ EIS) measurements were performed on NFMO and NFCMMOF at different voltages during the initial charge–discharge cycle. The equivalent circuit used for fitting is shown in Figure S34. As illustrated in Figure 5d,e and Figure S35a,b, the high‐frequency semicircles in the Nyquist plots exhibit pronounced differences, particularly in the high‐voltage region. NFMO displays the largest semicircle, indicating the highest charge‐transfer resistance (R_ct_) and sluggish interfacial kinetics, which can be primarily attributed to structural degradation and suppressed redox activity [51]. In contrast, NFCMMOF exhibits the smallest semicircle, corresponding to a markedly reduced R_ct_ and more efficient interfacial charge transfer [52, 53]. The initial R_ct_ values of NFMO and NFCMMOF are 519.4 and 203.5 Ω, respectively, and NFCMMOF maintains a relatively low resistance throughout the entire electrochemical cycle (Figure S36a,b). A slight increase in R_ct_ is observed during discharge due to Na^+^ reinsertion; nevertheless, NFCMMOF retains a low R_ct_ of 189.54 Ω even at 2.0 V, indicating stable charge‐transfer behavior over the full voltage window. By contrast, NFMO shows a continuous increase in R_ct_ upon charging, reaching a maximum value of 1526 Ω at 4.2 V, which is associated with unstable local electric fields and lattice distortions at high states of charge. During the subsequent discharge process (3.0‐2.0 V), a secondary R_ct_ peak emerges, which, when correlated with XPS, GCD, and CV results, suggests aggravated Jahn‐Teller distortions that hinder Na^+^ transport and deteriorate electrochemical kinetics.

To capture the potential‐dependent kinetic evolution, in situ EIS‐DRT analyses were performed on NFMO and NFCMMOF during both charging and discharging. NFMO exhibits pronounced voltage sensitivity and progressively dispersive relaxation behavior. During charging, the intermediate‐frequency contribution (log10(τ) ≈ −1 to 0, corresponding to τ ≈ 0.1‐1 s) is significantly intensified and broadened, accompanied by a substantial accumulation of slow processes at long relaxation times (log10(τ) ≈ 1–2, τ ≈ 10–100 s). This evolution manifests as an extended high‐intensity region in the DRT contour map at high states of charge (Figure 5f, h). The amplification and dispersion of the long‐τ contribution indicate that polarization dominated by sluggish transport and/or structural relaxation becomes increasingly pronounced at high voltages, consistent with interlayer gliding and lattice distortion associated with the P2‐O2 transition tendency [54]. During discharge, NFMO further shows a clear re‐emergence of enhanced mid‐to‐long τ components (notably within log10(τ) ≈ −1 to 1) in the 3.0–2.0 V range, evidencing incomplete kinetic reversibility and additional polarization coupled with structural hysteresis (e.g., Jahn–Teller related distortion), which hinders Na^+^ reinsertion (Figure S37). In contrast, NFCMMOF displays a much more constrained and reversible DRT evolution over the entire voltage window. At different potentials, the medium‐τ features remain narrower and highly overlapped, while long‐τ polarization (log10(τ) ≥ 1) is substantially suppressed, without the uncontrolled broadening observed in NFMO. Correspondingly, the contour maps reveal a reduced and more localized high‐intensity region at long τ during both charging and discharging (Figure 5g,i and Figure S38). These results demonstrate that Cu/Mg/F co‐doping effectively suppresses the development of slow polarization processes and stabilizes interfacial and transport kinetics throughout electrochemical cycling.

To further evaluate the practical applicability of NFCMMOF, a full sodium‐ion battery was assembled using NFCMMOF as the cathode and hard carbon (HC) as the anode. The operating mechanism of the NFCMMOF//HC full cell is illustrated in Figure 6a. To ensure stable cycling, the HC anode was pre‐sodiated prior to cell assembly, and the full cell was initially activated at 0.1 C. Figure 6b shows the galvanostatic charge–discharge (GCD) profiles of the NFCMMOF cathode (2.0–4.2 V) and HC anode (0.01–3.0 V) at 0.1 C. Notably, the GCD profiles of the full cell closely resemble those of the half‐cells, indicating consistent electrochemical behavior (Figure 6c). As shown in Figure 6d, the rate performance of the full cell was evaluated at 0.1, 0.2, 0.5, 1, 2, and 5 C, delivering discharge capacities of 93.3, 74.4, 61.0, 57.8, and 53.1 mAh·g^−1^, respectively. As illustrated in Figure 6e,f, the NFCMMOF full cell exhibits highly stable voltage profiles and excellent energy efficiency. At a low power density of 70.4 W·kg^−1^, it delivers a remarkable energy density of 281.5 Wh·kg^−1^. Even when the power density rises to 2260.69 W·kg^−1^, the energy density remains as high as 160.76 Wh·kg^−1^, demonstrating superior rate capability. Moreover, the cell maintains a high average operating voltage of 3.117 V at 70.4 W·kg^−1^, and this value remains almost unchanged even under high‐power conditions (Figure 6f), indicating negligible voltage degradation and outstanding electrochemical stability. Within the 2.0–4.2 V window, the full cell exhibits an initial discharge capacity of 75.4 mAh·g^−1^ at 1 C, retaining 84.09% after 200 cycles (Figure S39). At a high rate of 5 C, the full cell delivers 68.3 mAh·g^−1^ and maintains 79.70% capacity after 400 cycles (Figure 6g). As shown in Figure 6h, the NFCMMOF//HC full cell exhibits excellent electrochemical stability compared to the previously reported P2‐type layered oxide materials (Table S11). These results demonstrate the excellent rate capability and long‐term cycling stability of the NFCMMOF//HC full cell, highlighting its strong potential for practical sodium‐ion energy storage applications.

**FIGURE 6:**
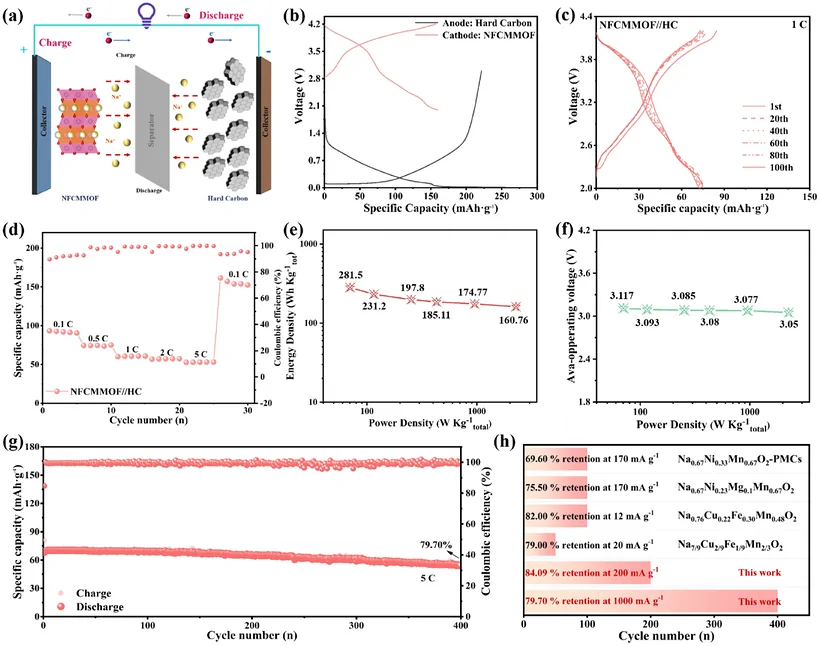
Performance of sodium‐ion full battery based on NFCMMOF and commercial hard carbon. (a) The full cell mechanism of NFCMMOF//HC. (b) The constant current charge‐discharge curves of NFCMMOF (2.0–4.2 V) and HC (0.01–3 V) at 20 mA·g^−1^ were obtained. (c) The GCD curve of NFCMMOF//HC in the first 100 cycles at 1 C. (d) The rate performance test of NFCMMOF//HC full cell at different current densities was carried out. (e) Diagram of the relationship between energy density and power density. (f) The relationship between average operating voltage and power density. (g) Long cycle test of NFCMMOF//HC full cell at 5 C. (h) Full battery performance comparison image.

## 3 Conclusion

In conclusion, we have developed a novel cross‐sublattice triple‐site co‐substitution strategy in P2‐type Na_0.67_Fe_0.08_Cu_0.25_Mn_0.62_Mg_0.05_O_1.95_F_0.05_ (NFCMMOF) to achieve precise covalent‐ionic balancing, simultaneously addressing the intrinsic challenges of P2‐type layered oxides including high‐voltage slab gliding, Jahn‐Teller distortion, lattice‐oxygen loss, and moisture‐induced degradation. This rational design not only stabilizes the TMO_2_ framework and oxygen sublattice but also shifts charge compensation toward a transition‐metal‐dominated, highly reversible Fe^3+^/Fe^4+^ redox process. As a result, NFCMMOF exhibits remarkable electrochemical performance, delivering high specific capacities of 162.9 and 88.9 mAh·g^−1^ at 0.1 and 10 C, excellent rate capability with 85.14% capacity retention at 10 C, and robust moisture tolerance. Mechanistic investigations combining in situ characterization and DFT calculations reveal that tri‐site co‐substitution expands the O‐Na‐O diffusion slab, reduces Na^+^ migration barriers, and lowers charge‐transfer resistance, facilitating rapid ion and electron transport. Impressively, the NFCMMOF full cell delivers high energy densities of 281.5 and 160.76 Wh·kg^−1^ at power densities of 70.4 and 2260.69 W·kg^−1^, respectively. Overall, this study introduces a versatile and rational covalent‐ionic balancing blueprint, providing a generalizable strategy to design high‐voltage, fast‐kinetic P2‐type cathodes for next‐generation sodium‐ion batteries.

## Author Contributions

H.X.: Investigation, Writing – original draft, Experimental Operation, Conceptualization, Writing – review & editing. X.S.: Validation. Z.L.: Validation. W.H.: Writing – review & editing, Project administration. G.Z.: Conceptualization, Writing – review & editing. Y.Z.: Writing – review & editing, Project administration.

## Conflicts of Interest

The authors declare no conflict of interest.

## Supporting information




**Supporting File**: advs76648‐sup‐0001‐SuppMat.docx.

## Data Availability

The data that support the findings of this study are available from the corresponding author upon reasonable request.

## References

[1] D. M. Zhou , C. Zeng , J. Xiang , T. Wang , Z. T. Gao , C. L. An , and W. X. Huang , “Review on Mn‐based and Fe‐based layered cathode materials for sodium‐ion batteries,” Ionics 28 (2022): 2029–2040, 10.1007/s11581-022-04519-1.

[2] Q. H. Gui , B. Xu , K. Yu , X. Y. Wang , J. Z. Li , Y. G. Xie , R. Yu , X. C. Zhou , and L. Mao , “Comparison of NaNi_1/3_Fe_1/3_Mn_1/3_O_2_ and Na_4_Fe_3_(PO_4_)_2_(P_2_O_7_) Cathode Sodium‐Ion Battery Behavior Under Overcharging Induced Thermal Runaway,” Chemical Engineering Journal 497 (2024): 154732, 10.1016/j.cej.2024.154732.

[3] P. A. Aparicio and N. H. de Leeuw , “Electronic Structure, Ion Diffusion and Cation Doping in The Na_4_ VO(PO_4_) 2 Compound As a Cathode Material for Na‐Ion Batteries,” Physical Chemistry Chemical Physics 22 (2020): 6653–6659, 10.1039/C9CP05559B. 32159169

[4] J. H. Wang , F. T. Xu , X. M. Fan , C. M. Zheng , Y. S. Zhao , L. L. Zuo , X. R. Yun , D. Lu , P. T. Xiao , and Y. F. Chen , “Preparation of Printing and Dyeing Sludge Biochar/Macromolecule Polymer Microsphere As a Non‐Sacrificial Persulfate Activator with Excellent Reusability and Reduced Heavy Metals Leaching,” Chemical Engineering Journal 500 (2024): 13.

[5] J. J. Han , Y. J. Lin , Y. Y. Yang , D. X. Zuo , C. P. Wang , and X. J. Liu , “Dominant Role of M Element on the Stability and Properties of Prussian Blue Analogues NaxMFe(CN)_6_ (M = 3d Transition Metal) as Cathode Material for the Sodium‐Ion Batteries,” Journal of Alloys and Compounds 870 (2021): 159533, 10.1016/j.jallcom.2021.159533.

[6] S. W. Xu , H. X. Chen , C. Li , R. H. Nie , Y. T. Yang , M. C. Zhou , X. Y. Zhang , and H. M. Zhou , “A New High‐Performance O_3_‐NaNi_0.3_Fe_0.2_Mn_0.5_O_2_ Cathode Material for Sodium‐Ion Batteries,” Ionics 29 (2023): 1873–1885, 10.1007/s11581-023-04963-7.

[7] J. X. Zhao , Y. S. Meng , D. M. Qi , and F. L. Zhu , “Structural Stability and Redox Activity Modulation of O_3_‐Type Layered Transition Metal Oxides by Lithium‐Ion Doping for High‐Performance Sodium‐Ion Batteries,” Ionics 31 (2025): 4321–4331, 10.1007/s11581-025-06259-4.

[8] D. M. Zhou , C. Zeng , D. Ling , T. Wang , Z. T. Gao , J. Li , L. L. Tian , Y. Wang , and W. X. Huang , “Sustainable alternative cathodes of sodium‐ion batteries using hybrid P_2_/O_3_ phase Na_0. 67_Fe_0. 5_Mn_0. 5− x_MgxO_2_ ,” Journal of Alloys and Compounds 931 (2022): 167567.

[9] Z. M. Zhou , Y. D. Qian , X. M. Chen , J. Chen , X. Z. Zhou , W. X. Kuang , X. Y. Shi , X. Q. Wu , L. Li , J. Z. Wang , and S. L. Chou , “Oxygen release suppression and electronic conductivity enhancement for high performance Li‐and Mn‐rich layered oxides cathodes by chalcogenide redox couple and oxygen vacancy generations,” Advanced Functional Materials 34, no. 15 (2024): 2310873.

[10] X. N. Li , X. Y. Tang , M. D. Zhang , M. Ge , X. J. Liu , Y. T. Cui , Y. W. Xu , H. S. Zhang , Y. H. Yin , and S. T. Yang , “Multiple Strategies to Build High‐Performance Spherical Na‐Ion Layered Oxide Cathodes,” Nano Letters 24 (2024): 14924–14931, 10.1021/acs.nanolett.4c02644. 39536130

[11] L. Gao , G. H. Chen , L. L. Zhang , B. Yan , and X. L. Yang , “Engineering Pseudocapacitive MnMoO_4_@C Microrods for High Energy Sodium Ion Hybrid Capacitors,” Electrochimica Acta 379 (2021): 138185, 10.1016/j.electacta.2021.138185.

[12] J. Peng , W. B. Hua , Z. Yang , J. Y. Li , J. S. Wang , Y. R. Liang , L. F. Zhao , W. H. Lai , X. Q. Wu , Z. X. Cheng , G. Peleckis , S. Indris , J. Z. Wang , H. K. Liu , S. X. Dou , and S. L. Chou , “Structural Engineering of Prussian Blue Analogues Enabling All‐Climate and Ultralong Cycling Sodium‐Ion Batteries,” ACS Nano 18 (2024): 19854. 10.1021/acsnano.4c07021 39007545

[13] F. Q. Lu , J. H. Wang , S. Q. Chang , L. H. He , M. X. Tang , Q. Wei , S. Y. Mo , and X. J. Kuang , “New‐type NASICON‐Na_4_FeV(PO_4_)_3_ cathode With high retention and durability for sodium ion batteries,” Carbon 196 (2022): 562–572, 10.1016/j.carbon.2022.05.033.

[14] J. X. Yu , H. F. Yu , L. L. Zhou , Q. L. Cheng , and H. Jiang , “, Trace Ti/Mg Co‐doped O_3_‐Type Layered Oxide Cathodes with Enhanced Kinetics and Stability for Sodium‐Ion Batteries,” Applied Surface Science 649, no. 7 (2024): 159127.

[15] M. R. Yang , S. P. Deng , S. S. Cheng , J. W. Zhao , S. Y. Li , and Y. Bai , “Unlocking Fast and Reversible Sodium Intercalation in Na_3_MnTi(PO_4_)_3_ Cathode toward High‐Performance Sodium‐Ion Batteries,” Nano Research 18, no. 9 (2025): 94907561.

[16] J. Peng , Y. Gao , H. Zhang , Z. G. Liu , W. Zhang , L. Li , Y. Qiao , W. S. Yang , J. Z. Wang , S. X. Dou , and S. L. Chou , “Ball Milling Solid‐State Synthesis of Highly Crystalline Prussian Blue Analogue Na_2_−xMnFe(CN)₆ Cathodes for All‐Climate Sodium‐Ion Batteries,” Angewandte Chemie International Edition 61, no. 10 (2022): 202205867. 10.1002/anie.202205867 35583767

[17] S. Y. Chu , Y. J. Zhong , K. M. Liao , and Z. P. Shao , “Layered Co/Ni‐Free Oxides for Sodium‐Ion Battery Cathode Materials,” Current Opinion in Green and Sustainable Chemistry 17 (2019): 29–34, 10.1016/j.cogsc.2019.01.006.

[18] Z. Zhang , C. J. M. Zhang , H. Q. Liu , Y. J. Gu , X. M. Xu , H. F. Wang , C. S. Zeng , Y. F. Wang , and F. Y. Chen , “Significant Improvement in Cycling and Rate Ability Originating From Regulation of Bonding and Transport Channel via Ba_2+_ Decoration in P_2_ layer Oxide Cathode,” Ceramics International 50 (2024): 12519–12528, 10.1016/j.ceramint.2024.01.161.

[19] Z. Mei , X. L. Li , C. Ma , J. Zeng , C. Y. Du , R. J. Luo , X. Xu , Z. Qian , Z. T. Zhou , Y. Zhang , Q. Cheng , Y. G. Fang , and Y. N. Zhou , “Copper‐Substituted P_3_‐type Na_0.54_ Mn_0.64_ Fe_0.16_ Mg_0.1_ Cu_0.1_ O_2_ Cathode Material for Sodium‐Ion Batteries With Enhanced Anionic Redox Reversibility,” Rare Metals 44 (2025): 2986–2996, 10.1007/s12598-024-03093-x.

[20] Y. You , L. H. Wang , Z. Li , S. W. Ou , J. Y. Xu , N. F. Yang , H. R. Luo , and M. L. Yuan , “Hierarchical Doping Engineering to Suppress P2–P2′ Phase Transition in Layered Sodium Cathodes for High‐Performance Sodium‐Ion Batteries,” Acta Materialia 298 (2025): 121383, 10.1016/j.actamat.2025.121383.

[21] Y. J. Park , J. U. Choi , J. H. Jo , C. H. Jo , J. Kim , and S. T. Myung , “A New Strategy to Build a High‐Performance P′ 2‐Type Cathode Material Through Titanium Doping for Sodium‐Ion Batteries,” Advanced Functional Materials 29, no. 10 (2019): 1901912.

[22] Z. M. Wang , H. Chen , Q. Zhao , Y. Shi , H. Y. Wang , Y. X. Ye , Y. Guo , Z. G. Du , and S. B. Yang , “Unveiling the Critical Role of Structural Water in Stabilizing Manganese‐Based Cathodes for High‐Performance Aqueous Zinc‐Ion Batteries,” Energy Storage Mater 71, no. 9 (2024): 103580.

[23] W. H. Qiu , Z. W. Chen , Z. Y. Liu , W. Xu , K. J. Zhang , Y. Hou , J. G. Lu , X. L. Zhan , Y. Y. Li , and Q. H. Zhang , “Rational Design of High‐Entropy Cathodes to Optimize Fast Charging Performance in Sodium‐Ion Batteries,” Advanced Functional Materials 35 (2025): 2422106, 10.1002/adfm.202422106.

[24] C. Peng , J. Zeng , Z. Yang , C. Zhang , F. Li , H. Wu , X. Tan , Z. Xiong , H. Yan , J. Wang , M. Zhu , S. Chen , and J. Liu , “Rigid Flexible Pillaring via Synergistic Co‐Doping Stabilizes High‐Capacity Sodium Layered Oxide Cathode,” Advanced Materials 38 (2026): 73078, 10.1002/adma.73078. 42059603

[25] Z. Liu , J. Wu , J. Zeng , F. Li , C. Peng , D. Xue , M. Zhu , and J. Liu , “Co‐Free Layered Oxide Cathode Material With Stable Anionic Redox Reaction for Sodium‐Ion Batteries,” Advanced Energy Materials 13 (2023): 2301471, 10.1002/aenm.202301471.

[26] X. Tan , J. Zeng , L. Sun , C. Peng , Z. Li , S. Zou , Q. Shi , H. Wang , and J. Liu , “Current issues and corresponding optimizing strategies of layered oxide cathodes for sodium‐ion batteries,” InfoMat 7 (2025): 12636.

[27] Z. W. Chen , M. L. Yang , G. J. Chen , G. X. Tang , Z. Y. Huang , M. H. Chu , R. Qi , S. M. Li , R. Wang , C. Q. Wang , T. L. Zhang , J. J. Zhai , W. G. Zhao , J. R. Zhang , J. Chen , L. H. He , J. P. Xu , W. Yin , J. Wang , and Y. G. Xiao , “Triggering Anionic Redox Activity in Fe/Mn‐Based Layered Oxide for High‐Performance Sodium‐Ion Batteries,” Nano Energy 94, no. 11 (2022): 106958.

[28] Y. X. Zhang , G. Q. Liu , Q. Sun , D. L. Qiao , J. G. Chen , L. Wen , and M. J. Zhao , “Synergistic Modulation of Crystal Structure and Interface Chemistry for Long‐Life High‐Voltage Sodium‐Ion Batteries,” Journal of Energy Storage 102, no. 26 (2024): 113396.

[29] Y. X. Cai , Z. J. Luo , K. S. Liao , X. Xin , M. J. Zhou , Y. J. Cheng , R. Liu , X. F. Yan , S. Papovic , K. Zheng , and K. Swierczek , “Dual‐site Zn doping boosts longevity and air stability of O_3_‐type NaNi_1/3_Fe_1/3_Mn_1/3_O_2_ cathode for high‐performance sodium‐ion batteries,” Journal of Power Sources 631 (2025): 236272, 10.1016/j.jpowsour.2025.236272.

[30] X. N. Li , M. Ge , M. D. Zhang , X. Y. Tang , X. J. Liu , Y. T. Cui , H. S. Zhang , Y. Yang , Y. H. Yin , and S. T. Yang , “Prilling and Coating Strategy to Synthesize High‐Performance Spherical NaNi_0.4_ Fe_0.2_ Mn_0.4_ O_2_ Cathode Materials for Sodium Ion Batteries,” Langmuir 40 (2024): 18610–18618, 10.1021/acs.langmuir.4c02065. 39172731

[31] X. Y. Zhou , X. Huang , Y. C. Cui , Y. Zhu , L. L. Wang , X. B. Wang , and S. C. Tang , “Cu‐Doped Spherical P_2_‐Type Na_0.7_ Fe_0.23–x_ Cu x Mn_0.77_ O_2_ Cathode for High‐Performance Sodium‐Ion Batteries,” ACS Applied Materials & Interfaces 16 (2024): 36354–36362, 10.1021/acsami.4c05516. 38955841

[32] C. Su , G. Liu , Q. Sun , L. Wen , Z. Chen , and M. Zhao , “New Strategy to Build a High‐Performance P′2‐Type Cathode Material Through Oxygen Vacancies and Mg Substitution for Sodium‐Ion Batteries,” ACS Applied Energy Materials 7 (2024): 1927–1937, 10.1021/acsaem.3c03039.

[33] Y. X. Kuang , Y. X. Wu , H. Y. Zhang , and H. P. Sun , “Interface issues of layered transition metal oxide cathodes for sodium‐ion batteries: Current status, recent advances, strategies, and prospects,” Molecules 29, no. 31 (2024): 5988. 10.3390/molecules29245988 PMC11677498 39770077

[34] X. Li , J. L. Xu , H. Y. Li , H. Zhu , S. H. Guo , and H. S. Zhou , “Synergetic Anion–Cation Redox Ensures a Highly Stable Layered Cathode for Sodium‐Ion Batteries,” Advancement of Science 9, no. 9 (2022): 2105280. 10.1002/advs.202105280 PMC9165485 35393768

[35] X. Wang , Z. Yang , D. Chen , B. Lu , Q. Zhang , Y. Hou , Z. Wu , Z. Ye , T. Li , and J. Lu , “Structural Regulation of P2‐Type Layered Oxide With Anion/Cation Codoping Strategy for Sodium‐Ion Batteries,” Advanced Functional Materials 35 (2025): 2418322, 10.1002/adfm.202418322.

[36] Q. C. Wang , J. K. Meng , X. Y. Yue , Q. Q. Qiu , Y. Song , X. J. Wu , Z. W. Fu , Y. Y. Xia , Z. Shadike , J. Wu , X. Q. Yang , and Y. N. Zhou , “Tuning P2‐Structured Cathode Material by Na‐Site Mg Substitution for Na‐Ion Batteries,” Journal of the American Chemical Society 141 (2019): 840–848, 10.1021/jacs.8b08638. 30562030

[37] Y. Yoda , K. Kubota , K. Kuroki , S. Suzuki , K. Yamanaka , T. Yaji , S. Amagasa , Y. Yamada , T. Ohta , and S. Komaba , “Elucidating Influence of Mg‐ and Cu‐Doping on Electrochemical Properties of O3‐Na x [Fe,Mn]O_2_ for Na‐Ion Batteries,” Small 16 (2020): 2006483, 10.1002/smll.202006483. 33230940

[38] R. Huang , S. H. Luo , Q. Sun , X. Yan , H. R. Zhang , L. X. Qian , X. Liu , X. Z. Cao , P. Zhang , and S. X. Yan , “Mn Vacancy Engineering Design of Fe/Mn Based Cathodes for Breaking the spell between “sodium limiting” in Air‐stabilization and "Sodium Promoting" on Kinetics,” Chemical Engineering Journal 499 (2024): 11.

[39] T. Yuan , P. Z. Li , Y. Y. Sun , H. Y. Che , Q. F. Zheng , Y. X. Zhang , S. Huang , J. Qiu , Y. P. Pang , J. H. Yang , Z. F. Ma , and S. Y. Zheng , “Refining O3‐Type Ni/Mn‐Based Sodium‐Ion Battery Cathodes via “Atomic Knife” Achieving High Capacity and Stability,” Advanced Functional Materials 35 (2025): 14.

[40] S. H. Jeong , I. K. Kim , S. Eom , H. Hwang , Y. H. Jung , and J. H. Kim , “Engineering the local chemistry through fe substitution in layered P2‐Na0. 7Ni0. 2Co0. 2Mn0. 6O_2_ for high‐performance Sodium‐Ion batteries,” Energy Storage Mater 75, no. 9 (2025): 104041.

[41] W. Chen , X. Y. Feng , Y. J. Wang , J. L. Chen , W. Yang , H. B. Zou , and S. Z. Chen , “P2‐Type Na₀.₆₇Fe₀._5_Mn₀._3_ _5_Co₀.₁_5_O_2_ Cathode Modified by an Active Coating Layer of K_2_Na(Co(NO_2_)₆) for Sodium‐Ion Batteries with Improved Cycling Stabilit,” Journal of Alloys and Compounds 970, no. 10 (2024): 172531.

[42] J. Xiao , H. Gao , Y. Xiao , S. J. Wang , C. Gong , Z. F. Huang , B. Sun , C. L. Dong , X. Guo , H. Liu , and G. X. Wang , “A Hydro‐Stable and Phase‐Transition‐Free P2‐Type Cathode with Superior Cycling Stability for High‐Voltage Sodium‐Ion Batteries,” Chemical Engineering Journal 506, no. 10 (2025): 160010.

[43] J. L. Qiu , M. L. Qin , F. Huang , Q. Cheng , S. Guo , X. X. Cao , Y. P. Lei , S. Q. Liang , and G. Z. Fang , “High‐Entropy Configuration Regulating Interlayer Oxygen Charge Toward High‐Voltage and Air‐Stability Layered Cathode in High‐Loading Sodium Ion Full Batteries,” Advanced Functional Materials 35 (2025): 11.

[44] M. M. Yan , K. Xu , Y. X. Chang , Z. Y. Xie , and S. L. Xu , “Cu/Ti co‐Doping Boosting P2‐Type Fe/Mn‐Based layered Oxide Cathodes for High‐Performance Sodium Storage,” Journal of Colloid and Interface Science 651 (2023): 696–704, 10.1016/j.jcis.2023.07.195. 37562311

[45] T. T. Yang , Y. L. Huang , J. Zhang , H. Zhu , J. C. Ren , T. Y. Li , L. C. Gallington , S. Lan , L. G. Yang , and Q. Liu , “Insights Into Ti doping for stabilizing the Na_2/3_Fe_1/3_Mn_2/3_O_2_ cathode in sodium ion battery,” Journal of Energy Chemistry 73 (2022): 542–548, 10.1016/j.jechem.2022.06.016.

[46] A. Ghosh , D. Bhattacharya , and P. Senguttuvan , “Synergistic Effect of (K + +Li + ) Cosubstitution on P2‐Type Fe–Mn Oxide Cathodes for Sodium‐ion Batteries,” ACS Applied Energy Materials 7 (2024): 9616–9624, 10.1021/acsaem.4c01699.

[47] X. T. Wang , Q. H. Zhang , C. Zhao , H. F. Li , B. D. Zhang , G. F. Zeng , Y. L. Tang , Z. Y. Huang , I. Hwang , H. T. Zhang , S. Y. Zhou , Y. F. Qiu , Y. G. Xiao , J. Cabana , C. J. Sun , K. Amine , Y. Sun , Q. S. Wang , G. L. Xu , L. Gu , Y. Qiao , and S. G. Sun , “Achieving a high‐performance sodium‐ion pouch cell by regulating intergrowth structures in a layered oxide cathode With anionic redox,” Nature Energy 9 (2024): 184–196, 10.1038/s41560-023-01425-2.

[48] Q. Ni , Y. Zhao , X. Yuan , J. Li , and H. Jin , “Dual‐Function of Cation‐Doping to Activate Cationic and Anionic Redox in a Mn‐Based Sodium‐Layered Oxide Cathode,” Small 18 (2022): 2200289, 10.1002/smll.202200289. 35585688

[49] Y. Zhang , Z. Li , Z. Li , W. Sun , X. Yuan , H. Jin , and Y. Zhao , “Electron Donor Enabling Mn‐Fe Based Layer Oxide Cathode With Durable Sodium Ion Storage,” Journal of Energy Chemistry 109 (2025): 740–748, 10.1016/j.jechem.2025.06.008.

[50] X. B. Song , R. A. Liu , J. T. Jin , X. D. Zhao , Y. Wang , Q. Y. Shen , Z. Q. Sun , X. H. Qu , L. F. Jiao , and Y. C. Liu , “Modulating the Oxygen Redox Activity of an Ultra‐High‐Capacity P3‐Type Cathode for Sodium‐Ion Batteries via Beryllium Introduction,” Energy Storage Mater 69, no. 10 (2024): 103368.

[51] C. Guo , X. Y. Fan , M. Liu , S. W. Xu , G. X. Wei , Z. B. Chen , Z. K. Guan , Y. Xiao , H. Xin , and P. F. Wang , “Amorphous Protective Layers to Reshape Inorganic‐Rich Interphases for High‐Voltage Sodium‐Ion Batteries,” Carbon Energy (2025): 70037, 10.1002/cey2.70037.

[52] J. D. Zhang , Z. S. Yu , Y. B. Zhu , J. Y. Cai , M. Q. Wang , P. K. Gao , Y. L. Zhang , N. Q. Zhang , D. Y. Wang , Y. Shen , and M. K. Wang , “Configuration Design and Interface Reconstruction to Realize the Superior High‐Rate Performance for Sodium Layered Oxide Cathodes,” Advanced Energy Materials 15, no. 10 (2025): 2405951.

[53] P. Zhang , G. H. Zhang , Y. K. Liu , Y. X. Fan , X. Y. Shi , Y. M. Dai , S. W. Gong , J. R. Hou , J. W. Ma , Y. H. Huang , and R. Y. Zhang , “Constructing P2/O_3_ Biphasic Structure of Fe/Mn‐Based Layered Oxide Cathode for High‐Performance Sodium‐Ion Batteries,” Journal of Colloid and Interface Science 654 (2024): 1405–1416, 10.1016/j.jcis.2023.10.129. 37918099

[54] Z. X. Huang , T. Q. Yang , J. M. Cao , K. Y. Zhang , Y. Liu , B. J. Xin , K. Xu , Y. Liu , X. Y. Zhou , J. Z. Guo , T. Wang , H. Geng , and X. L. Wu , “Multifunctional and Radii‐Matched High‐Entropy Engineering Toward Locally‐Regulable Metal Oxide Layers in Sodium‐Layered Oxide Cathode,” Angewandte Chemie International Edition 64 (2025): 202505367, 10.1002/anie.202505367. 40503599

