# Supplementary material for: Synergizing Covalent‐Ionic Tri‐Site Coordination in P2‐type Na0.67Fe0.33Mn0.67O2 Induces Suppressed Layer‐Slipping and Stablized Lattice Oxygen for Sodium‐Ion Batteries at Wide Potential
Source: Adv Sci (Weinh). 2026 Jul 21:e76648. Online ahead of print. doi: 10.1002/advs.76648 (PMC13387034; doi:10.1002/advs.76648)
Supplement: Supplementary file 1 — Supporting File: advs76648‐sup‐0001‐SuppMat.docx. [file ADVS-9999-e76648-s001.docx]

**Synergizing Covalent-Ionic Tri-Site Coordination in P2-type Na_0.67_Fe_0.33_Mn_0.67_O_2_ Induces Suppressed Layer-Slipping and Stablized Lattice Oxygen for Sodium-Ion Batteries at Wide Potential**

Haitao Xue^1^, Xudong Shi^1^, Zhiyue Lian^1^, Wenxiu He^1,^ *, Genqiang Zhang^2,^ *, Yongqiang Zhang^1,^ *

^1^School of Chemistry and Chemical Engineering, Inner Mongolia University of Science & Technology, Baotou, Inner Mongolia 014010, China

^2^Hefei National Research Center for Physical Sciences at the Microscale, Department of Materials Science and Engineering, University of Science and Technology of China, Hefei, Anhui 230026, China.

*Corresponding authors. E-mail addresses: wenxiu_he@foxmail.com (W. X. He), gqzhangmse@ustc.edu.cn (G.Q. Zhang), yongqiang_zhang1203@foxmail.com (Y.Q. Zhang).

**Methods**

**Materials**

Citric acid (99.5 %, Shanghai Macklin Biochemical Technology Co., Ltd.), anhydrous NaNO_3_ (99 %, Tianjin Fengchuan Chemical Reagent Co., Ltd.), Fe(NO_3_)_3_·9H_2_O (98.5 %, Shanghai Macklin Biochemical Technology Co., Ltd.), Mn(CH_3_COO)_2_·4H_2_O (99 %, Shanghai Yien Chemical Technology Co., Ltd.), Cu(NO_3_)_2_·3H_2_O (99 %, Shanghai Macklin Biochemical Technology Co., Ltd.)， Mg(NO_3_)_2_·6H_2_O (99 %, Shanghai Macklin Biochemical Technology Co., Ltd.), NaF (99 %, Sinopharm Chemical Reagent Co., Ltd).

**Synthesis of Na_0.67_Fe_0.08_Cu_0.25_Mn_0.62_Mg_0.05_O_1.95_F_0.05_**

Na_0.67_Fe_0.08_Cu_0.25_Mn_0.62_Mg_0.05_O_1.95_F_0.05_ was synthesized via a facile sol-gel method to ensure homogeneous mixing of the precursor ions at the molecular level. Citric acid was used as a chelating agent, while anhydrous NaNO_3_, Fe(NO_3_)_3_·9H_2_O, Mn(CH_3_COO)_2_·4H_2_O, Cu(NO_3_)_3_·3H_2_O, Mg(NO_3_)_2_·6H_2_O, and NaF were employed as the sources of Na, Fe, Mn, Cu, Mg, and F, respectively. To compensate for possible sodium volatilization during high-temperature calcination, 5 wt% excess Na was introduced. The molar ratio of Na: Fe: Mn: Cu: Mg: F was maintained at 7.5: 0.8: 6.2: 2.5: 0.5: 0.5 mmol. Typically, stoichiometric amounts of the above precursors were dissolved in distilled water under continuous magnetic stirring for 30 min to obtain a clear and homogeneous solution. Subsequently, citric acid was added with a citric acid/total metal ion molar ratio of 1: 1, which promoted uniform complexation of metal cations and suppressed compositional segregation during gel formation. The mixed solution was further stirred for 1 h and then heated in a water bath to gradually evaporate the solvent until a viscous gel was formed. The obtained gel precursor was vacuum-dried at 120 °C for 15 h to remove residual water and enhance precursor homogeneity. After drying, the xerogel was ground into a fine powder and pre-calcined at 450 °C for 5 h in air with a heating rate of 5 °C min^−1^ to decompose organic species, nitrates, and acetate residues. The pre-calcined powder was subsequently reground thoroughly to improve particle uniformity, pressed into pellets under 10 MPa to facilitate solid-state diffusion, and finally sintered at 900 °C for 10 h in air at a heating rate of 5 °C min^−1^. This high-temperature treatment enabled crystallization of the P2-type layered oxide phase and promoted homogeneous incorporation of Cu, Mg, and F into the lattice framework. After calcination, the products were naturally cooled to room temperature and then ground into fine powders for subsequent structural characterization and electrochemical measurements. The corresponding control samples were prepared using the same synthetic procedure by adjusting the precursor ratios and omitting the relevant dopant sources. The overall synthesis route and battery assembly process are schematically illustrated in Scheme 1 of the Supporting Information.

**Electrochemical measurements**

The cathode slurry was prepared by thoroughly mixing the active material (70 wt%), conductive carbon black (20 wt%), and polyvinylidene fluoride (PVDF, 10 wt%) in N-methyl-2-pyrrolidone (NMP) solvent, followed by homogeneous dispersion using a planetary ball mill. The obtained slurry was uniformly coated on aluminum foil and subsequently vacuum-dried at 80 °C for 12 h to remove the residual solvent. Circular electrodes with a diameter of ~10 mm were then punched and compacted under a pressure of 9 MPa. The mass, diameter, and thickness of the cathode active layer were approximately 1.2 ± 0.3 mg, 10 mm, and 18 μm, respectively. Metallic sodium foil, protected by a removable film to prevent oxidation, served as both the counter and reference electrode. The CR2025 coin-type cells were assembled in an argon-filled glovebox (H_2_O and O_2_ < 0.01 ppm), using Whatman GF/F glass fiber as the separator. The electrolyte consisted of 1 mol·L^−1^ NaClO_4_ dissolved in a mixed solvent of ethylene carbonate (EC), dimethyl carbonate (DMC), and ethyl methyl carbonate (EMC) in a 1: 1: 1 volume ratio, with an additional 2 vol% fluoroethylene carbonate (FEC) additive. For full-cell assembly, the NFM-CZ composite was employed as the cathode and commercial hard carbon (HC) as the anode. Prior to assembly, the HC electrode was pre-sodiated at a current density of 20 mA·g^−1^ within a voltage window of 0.01-3.0 V to ensure stable cycling performance.

Long-term cycling and rate capability tests were performed at room temperature using a LAND battery testing system within a voltage range of 2.0-4.2 V. Cyclic voltammetry (CV) and in-situ electrochemical impedance spectroscopy (EIS) measurements were performed on a CHI760E electrochemical workstation. The CV scans were recorded within a potential range of 2.0-4.2 V, while the EIS spectra were collected over a frequency range of 100 kHz to 10 mHz. In-situ EIS tests were conducted under a stepwise potential increment of 0.1 V, with a relaxation time of 5 min at each voltage step to ensure equilibrium. The galvanostatic intermittent titration technique (GITT) was also carried out using the LAND battery test system. During the GITT measurement, the coin cells were subjected to a constant current pulse of 20 mA·g^−1^ for 30 min, followed by an open-circuit relaxation period of 30 min to allow for complete Na^+^ diffusion equilibration.

**Calculation and analysis of GITT**

If the Eτ as a function of τ is linear, the Na^+^ diffusion coefficient can then be calculated by simplified Equation (1):

$\text{D}_{\text{Na}^{\text{+}}}\text{=}\frac{\text{4}}{\text{πτ}}\left( \frac{\text{m}_{\text{B}}\text{V}_{\text{M}}}{\text{M}_{\text{B}}\text{A}} \right)^{\text{2}}\left( \frac{\text{∆E}_{\text{S}}}{\text{∆}\text{E}_{\text{τ}}} \right)^{\text{2}}$ (1)

Where $D_{{Na}^{+}}$ (cm^2^·s^−1^) means the chemical diffusion coefficient, V_M_ (cm^3^·mol^−1^), the molar volume, weight, and molar weight of the active materials are indicated by m_B_, M_B_, and V_M_, respectively. A and τ (s) represent the surface area of the electrode and the testing time in each step, and ΔE_s_, ΔE_τ_ are the quasiequilibrium potential and the change of cell voltage E during the current pulse, respectively.

**Materials characterization**

The crystal structures of the samples were analyzed by X-ray diffraction (XRD, Ultima IV-185) using Cu Kα radiation (λ = 1.5405 Å) over 2θ = 10°-90°. Morphology and elemental distribution were examined by scanning electron microscopy (SEM, HITACHI SU8010) equipped with energy-dispersive X-ray spectroscopy (EDS, Ultim Max 80). The microstructure was further investigated by high-resolution transmission electron microscopy (HRTEM, JEM-F200) operated at 200 kV.

X-ray photoelectron spectroscopy (XPS, Thermo Fisher ESCALAB 250Xi) was employed to determine the valence states of elements in pristine and cycled electrodes, with binding energies calibrated to the C 1s peak at 284.8 eV. To probe structural, morphological, and valence evolution during cycling, ex-situ XPS and SEM analyses were performed on electrodes retrieved after selected charge-discharge cycles. All samples were handled in an argon-filled glovebox and stored under vacuum to prevent oxidation. In-situ XRD of half-cells was carried out using a custom fixture with a beryllium window, allowing real-time monitoring of structural changes during electrochemical cycling.

**Moisture resistance test**

The sample powder and the prepared electrode were exposed to a laboratory environment at room temperature of 25 ± 1 °C and humidity of RH ≈ 45-55 %.

**DFT calculation**

The density functional theory (DFT) calculations were carried out with the MS code^[1]^. The Perdew-Burke-Ernzerhof (PBE) functional within generalized gradient approximation (GGA)^[2]^ was used to process the exchange-correlation, while the projector augmented-wave pseudopotential (PAW)^[3]^ was applied with a kinetic energy cut-off of 500 eV, which was utilized to describe the expansion of the electronic eigenfunctions. The vacuum thickness was set to be 50 Å to minimize interlayer interactions. The Brillouin-zone integration was sampled by a Γ-centered 10 × 10 × 1 Monkhorst-Pack k-point. All atomic positions were fully relaxed until energy and force reached a tolerance of 1 × 10^−6^ eV and 0.01 eV/Å, respectively. The dispersion corrected DFT-D method was employed to consider the long-range interactions^[4]^.


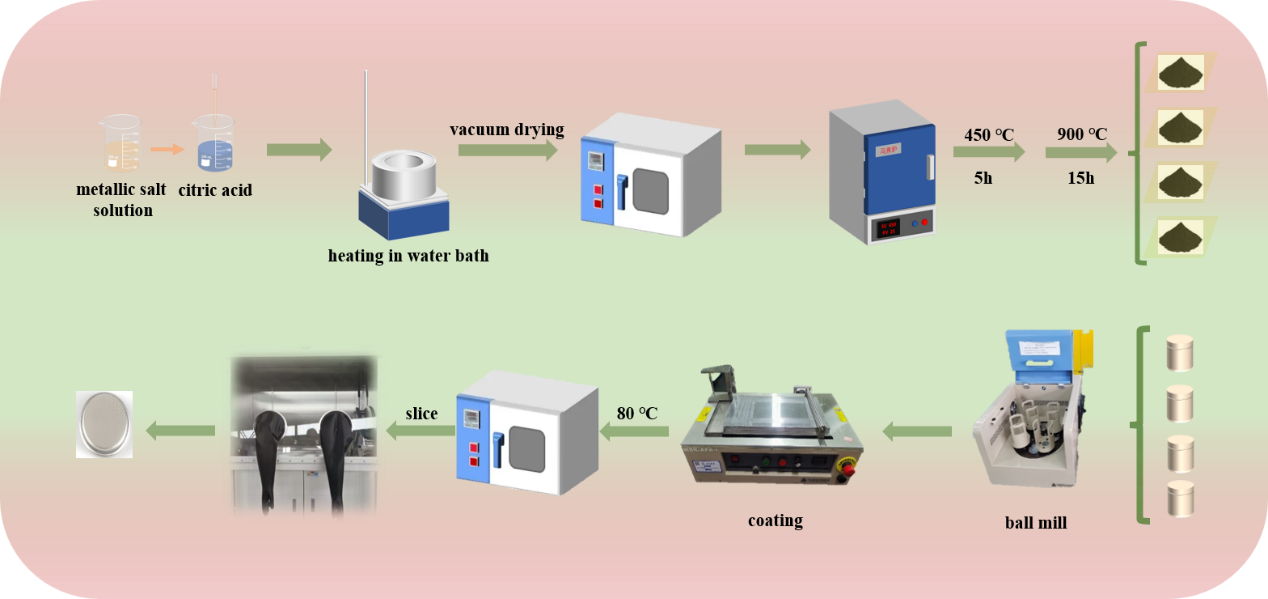


**Scheme 1.** Schematic diagram of synthesis process of Na_0.67_Fe_0.08_Cu_0.25_Mn_0.62_Mg_0.05_O_1.95_F_0.05._

**
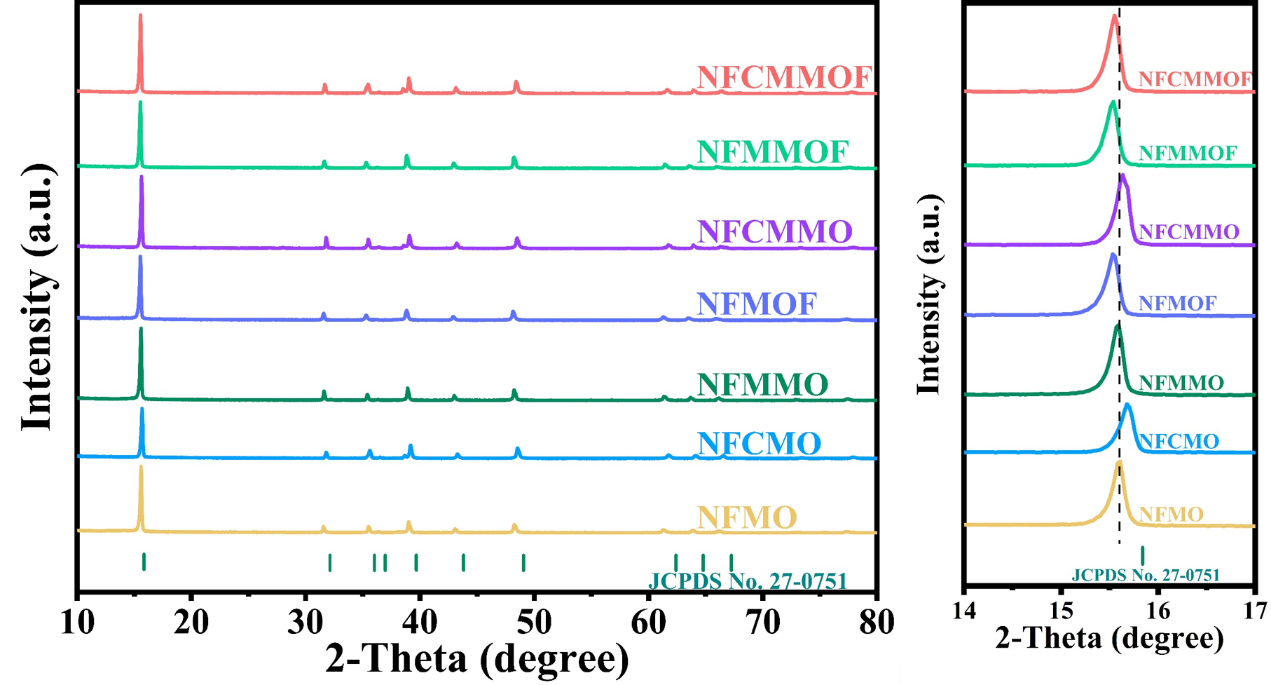
**

**Figure S1.** XRD patterns of the NFMO, NFCMO, NFMMO, NFMOF, NFCMMO, NFMMOF, and NFCMMOF.

**
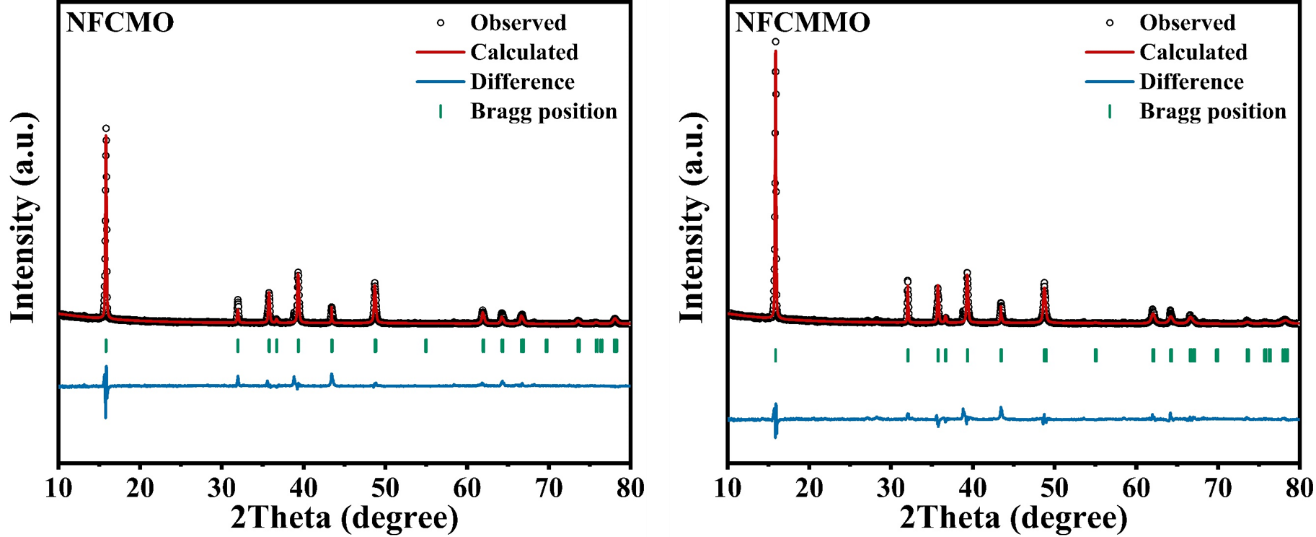
**

**Figure S2.** Rietveld refined XRD images: NFCMO; NFCMMO.

**
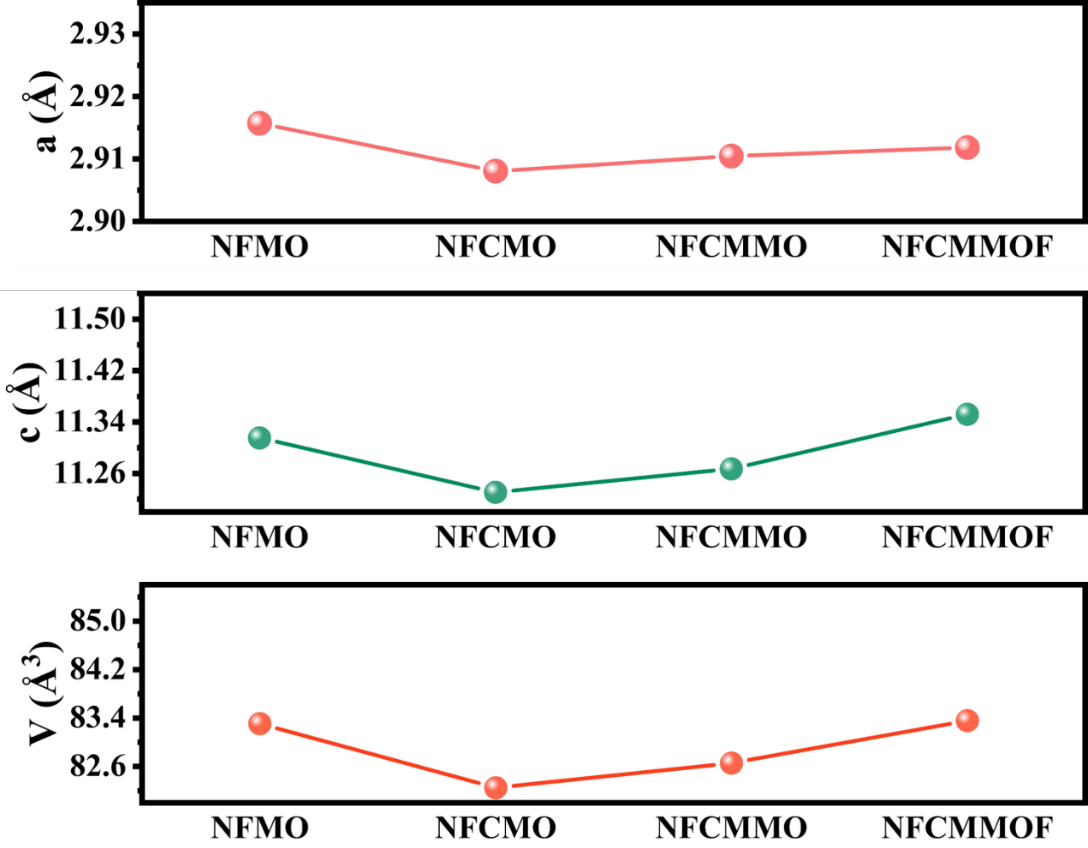
**

**Figure S3.** Comparison of the cell parameters of each material.


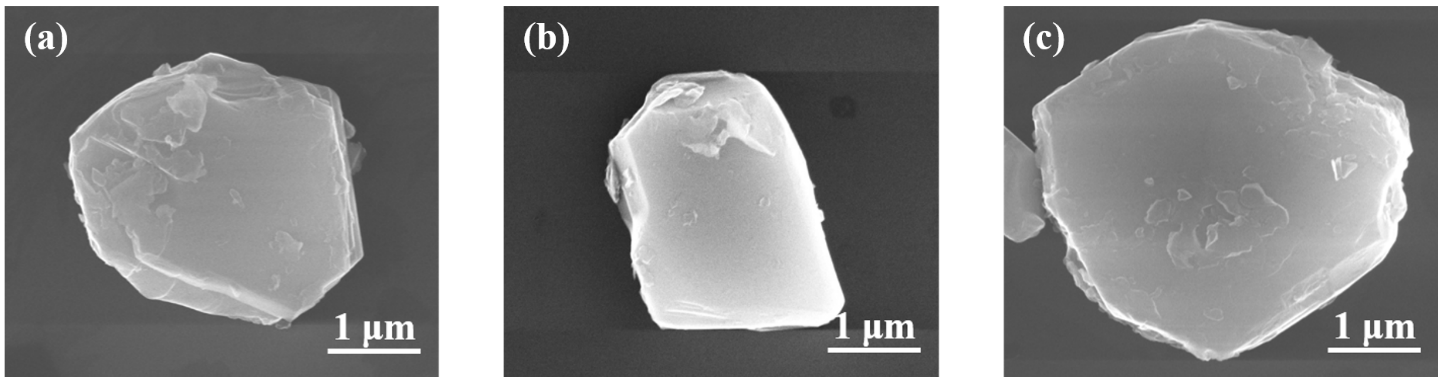


**Figure S4.** SEM images under 1 μm magnification: (a) NFMO; (b) NFCMO; (c) NFCMMO.

**
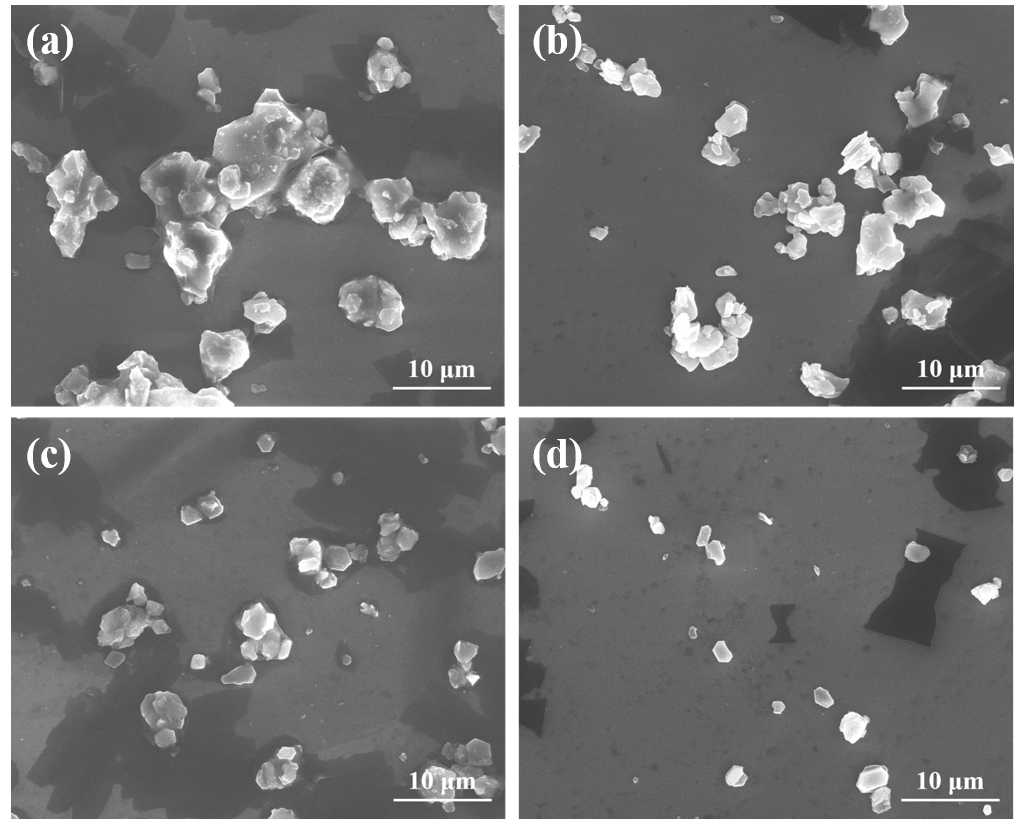
**

**Figure S5.** SEM images under 10 μm magnification: (a) NFMO; (b) NFCMO; (c) NFCMMO; (d) NFCMMOF.

**
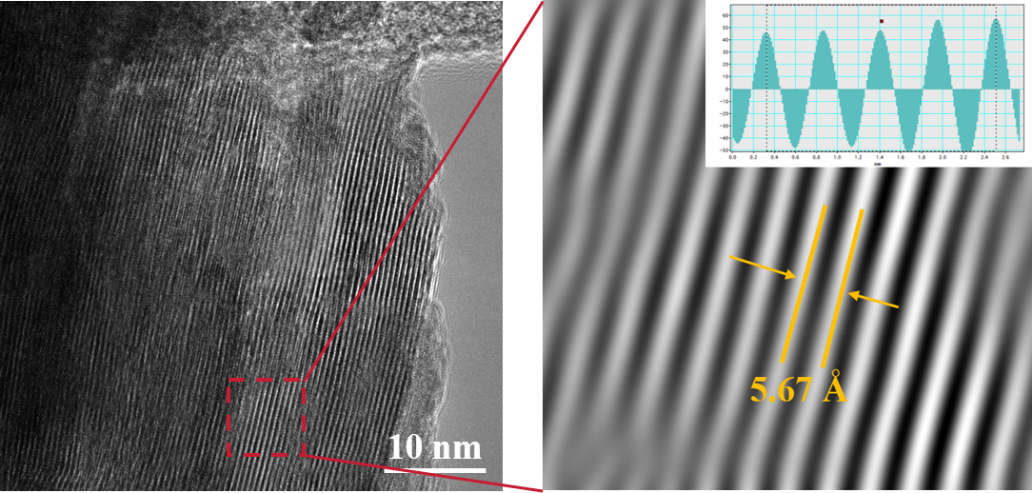
**

**Figure S6.** TEM images of NFMO.

**
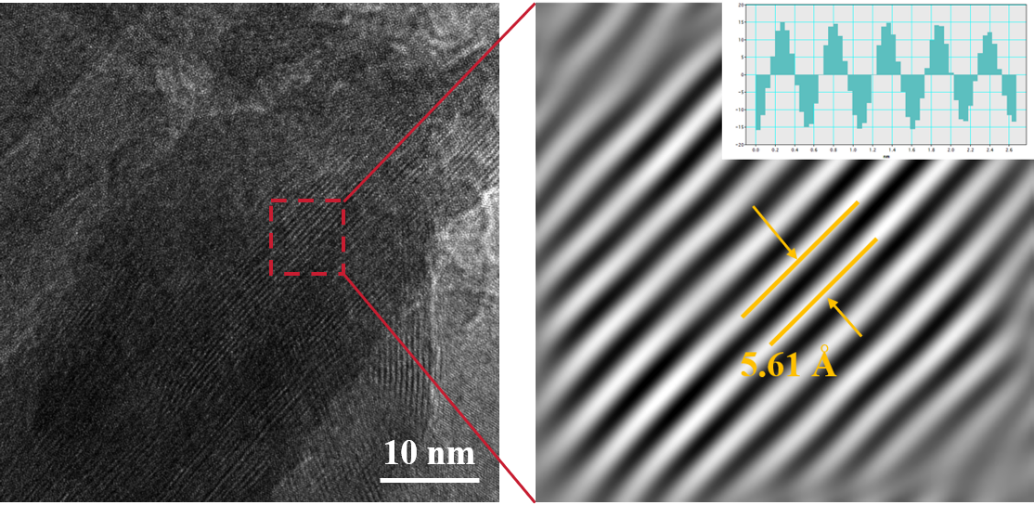
**

**Figure S7.** TEM images of NFCMO.


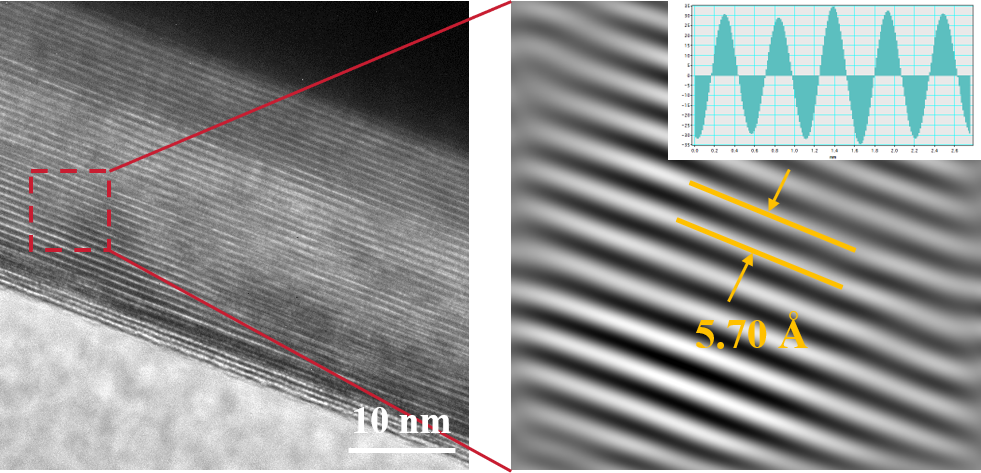


**Figure S8.** TEM images of NFCMMO.

**
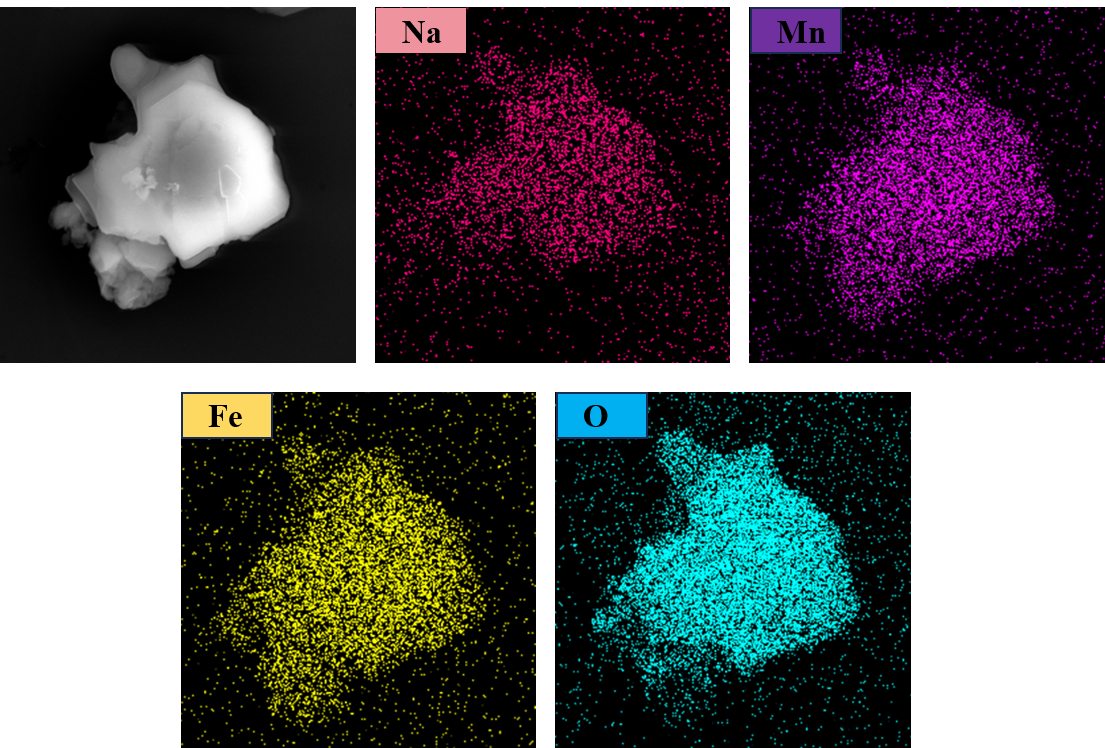
**

**Figure S9.** EDS images of NFMO.

**
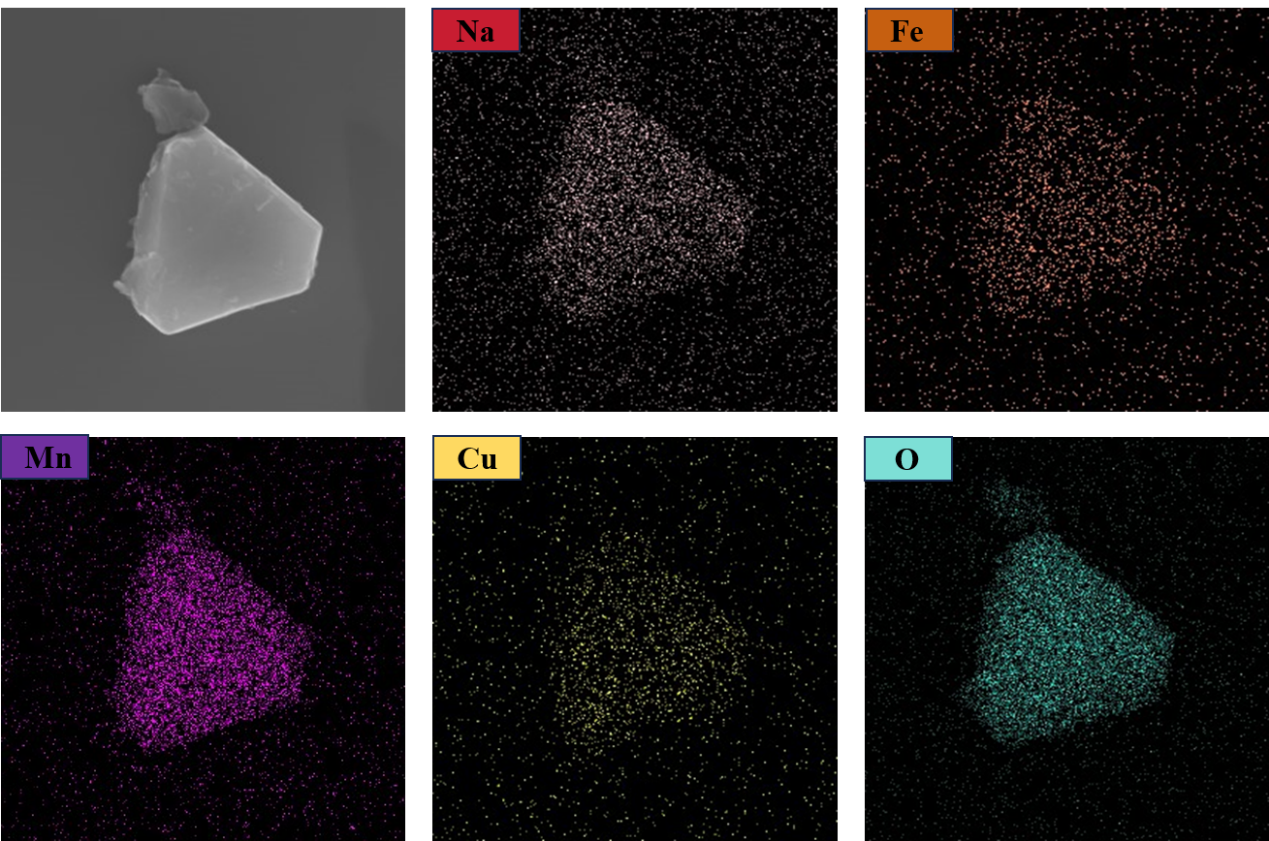
**

**Figure S10.** EDS images of NFCMO.

**
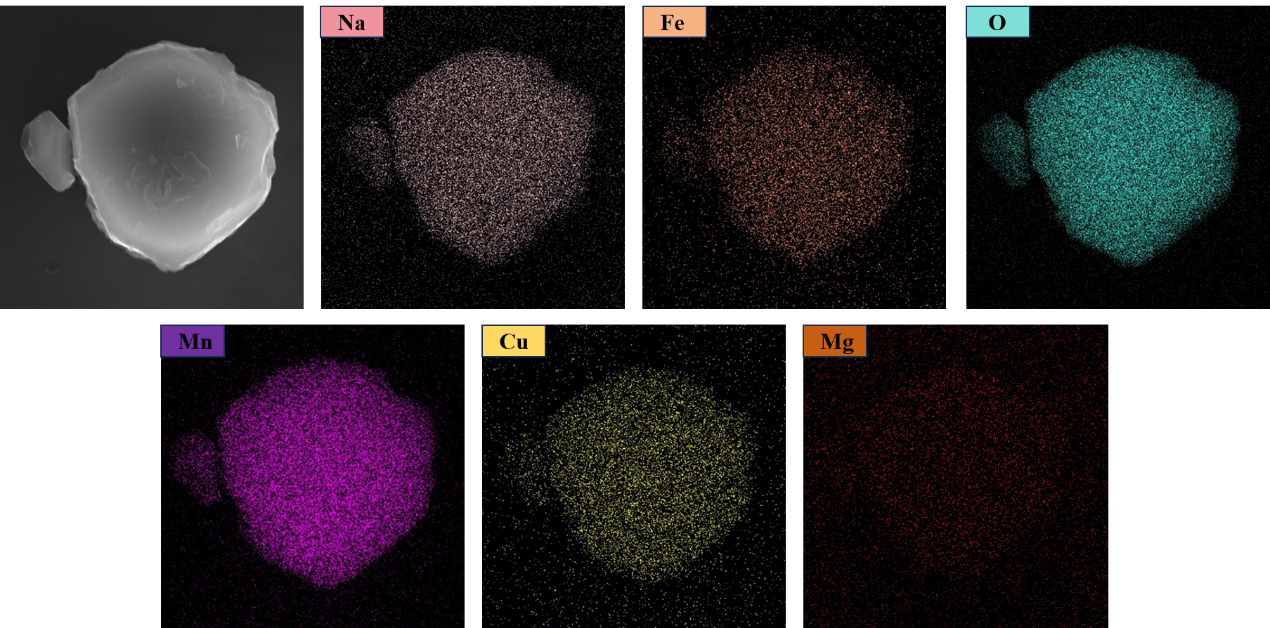
**

**Figure S11.** EDS images of NFCMMO.

**
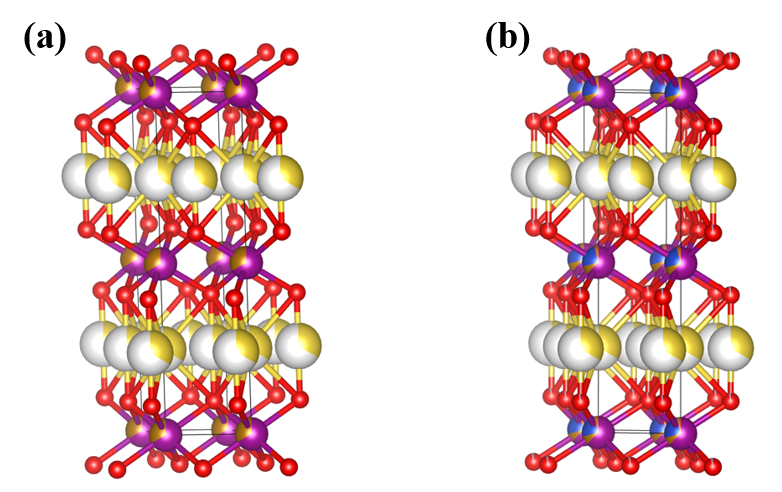
**

**Figure S12.** Theoretical calculation of the optimized structural model: (a) NFMO; (b) NFCMMOF.

**
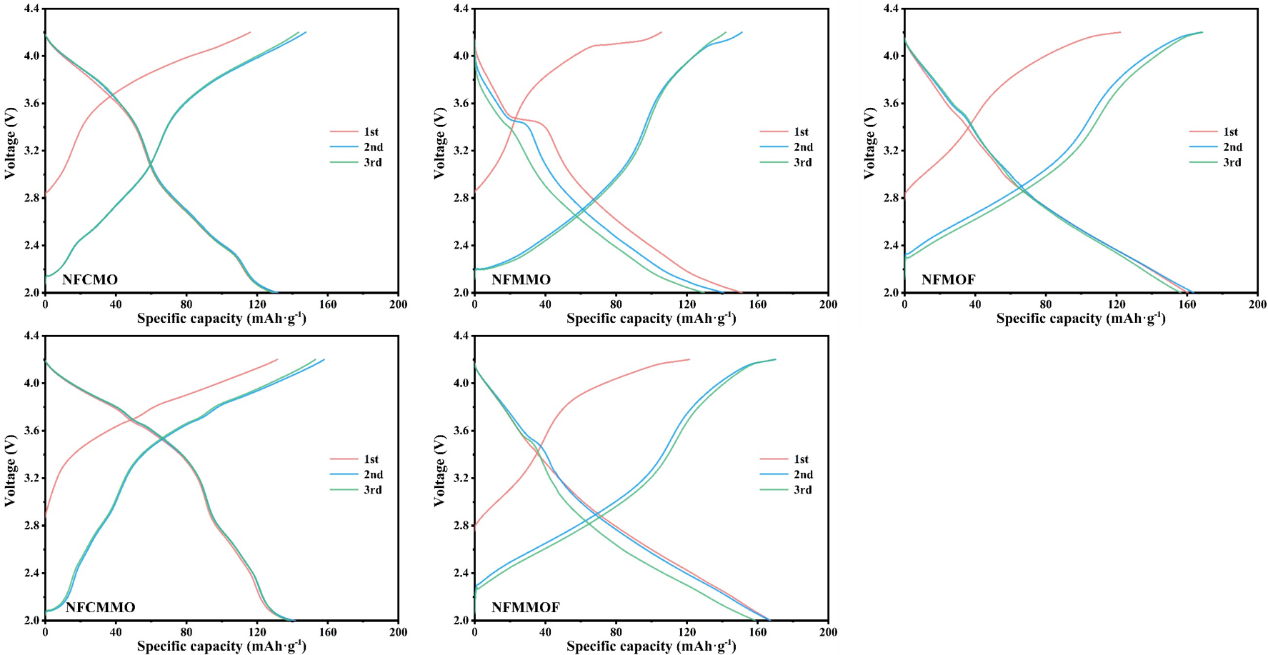
**

**Figure S13.** The GCD curves of the first three cycles of NFCMO, NFMMO, NFMOF, NFCMMO and NFMMOF at 0.1 C rate.

**
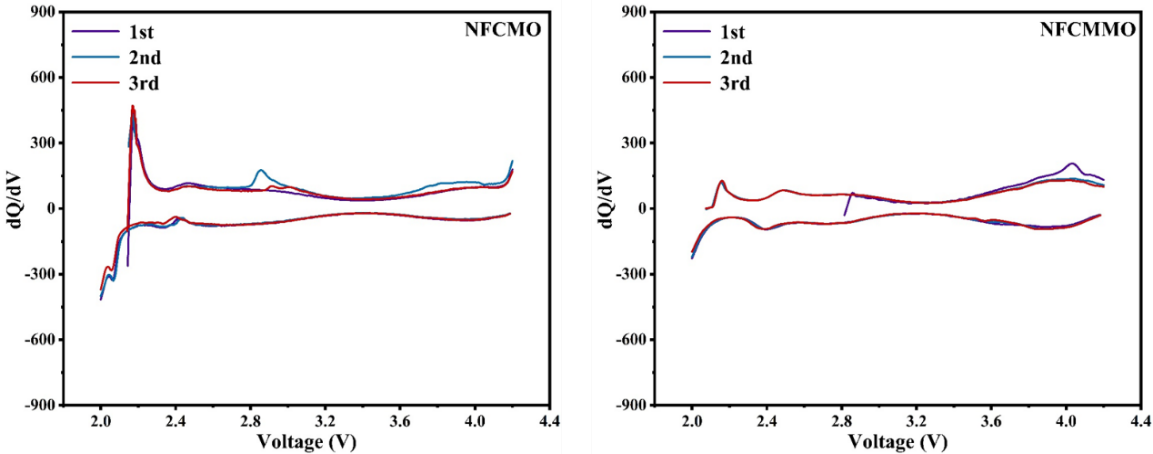
**

**Figure S14.** The differential capacity curves of NFCMO and NFCMMO in the first three cycles at 0.1 C rate.

**
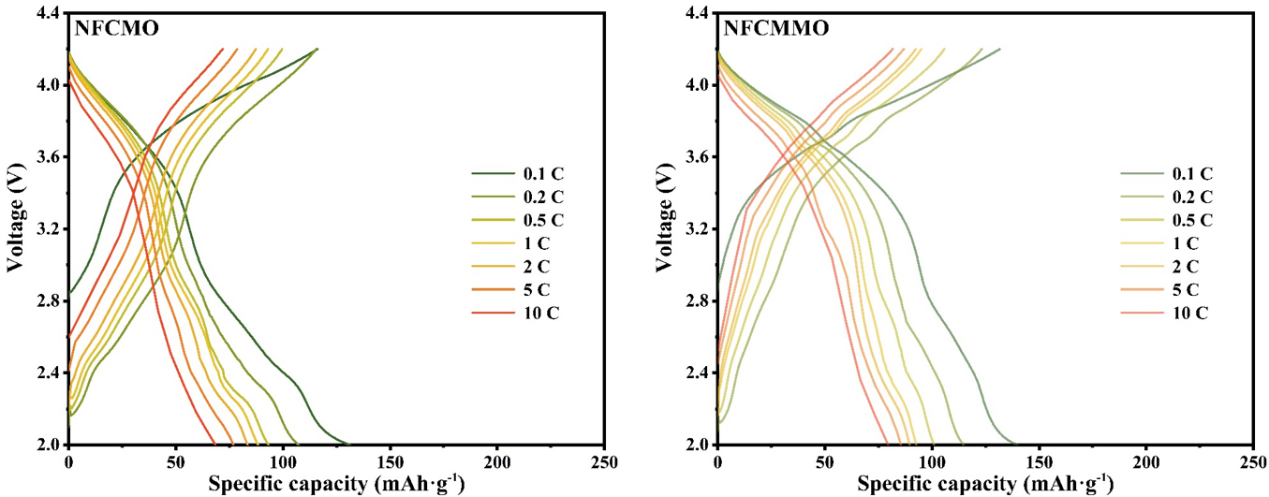
**

**Figure S15.** The GCD curves of NFCMO and NFCMMO at different rates.

**
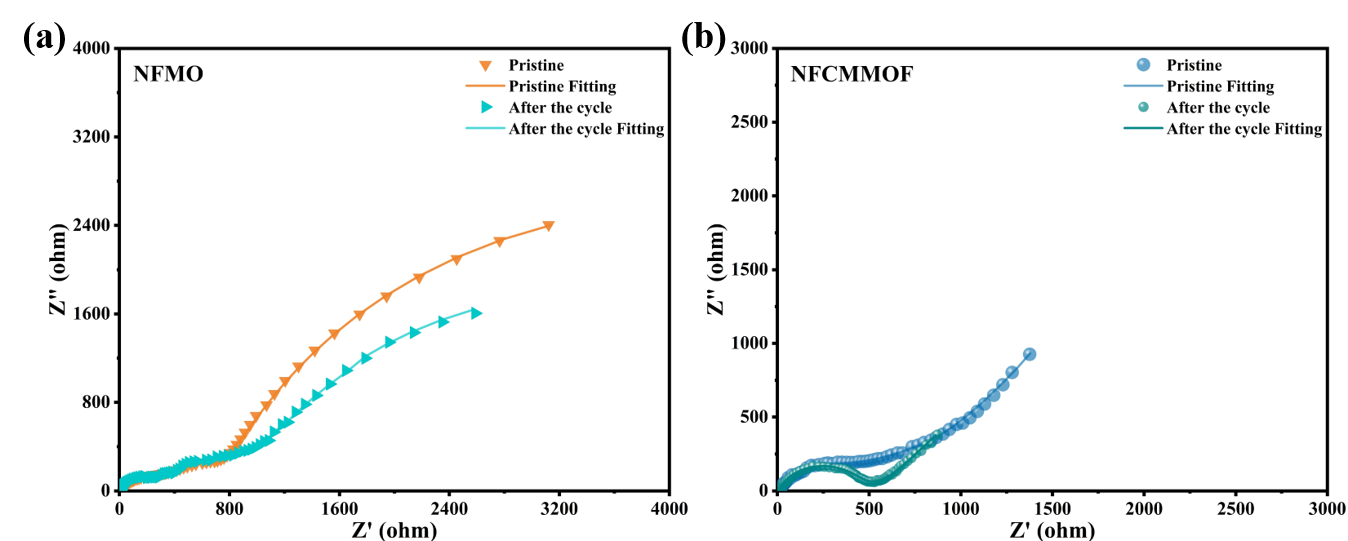
**

**Figure S16.** EIS test curve after rate cycling: (a) NFMO; (b) NFCMMOF.

**
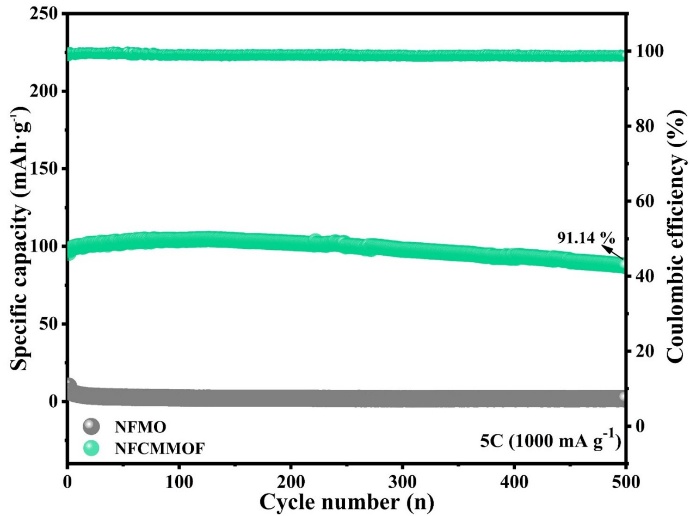
**

**Figure S17.** Long cycle test performance at 5 C rate.

**
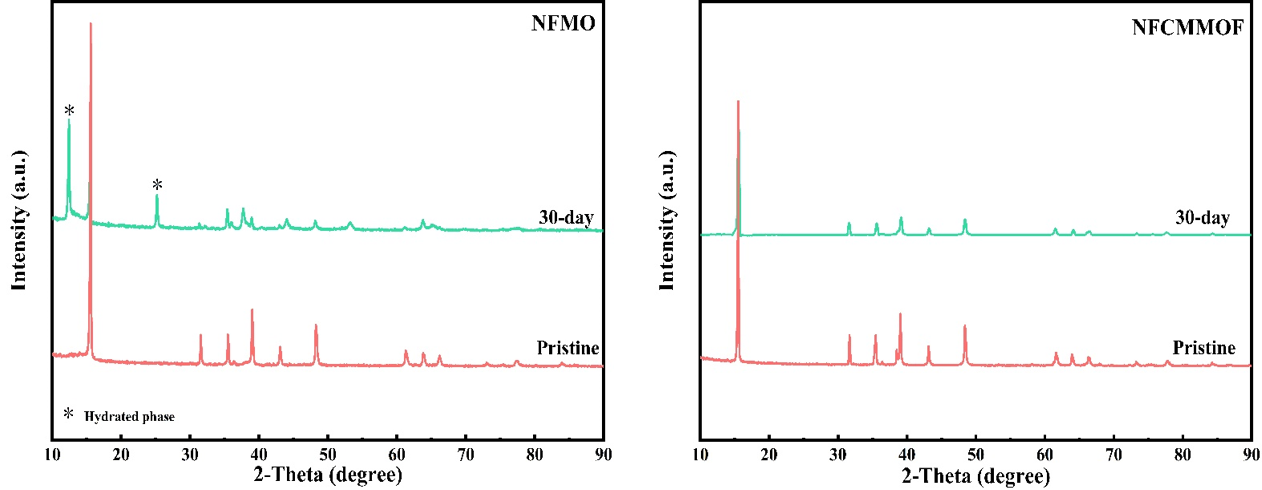
**

**Figure S18.** XRD test images were performed after NFMO and NFCMMOF were exposed to humid air for 30 days.

**
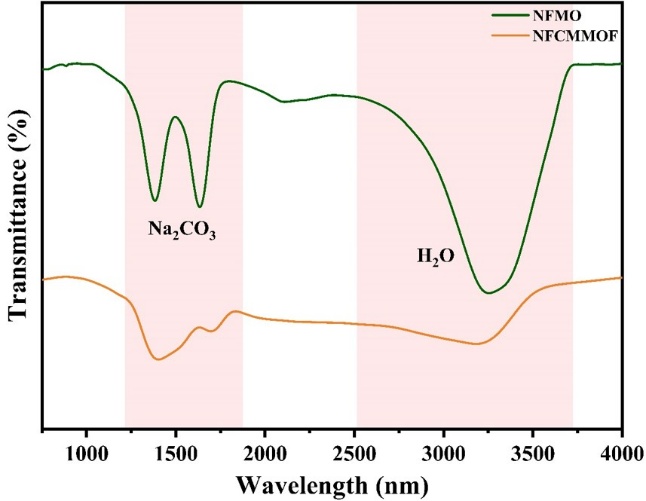
**

**Figure S19.** Infrared spectrum test images of NFMO and NFCMMOF after exposure treatment.

**
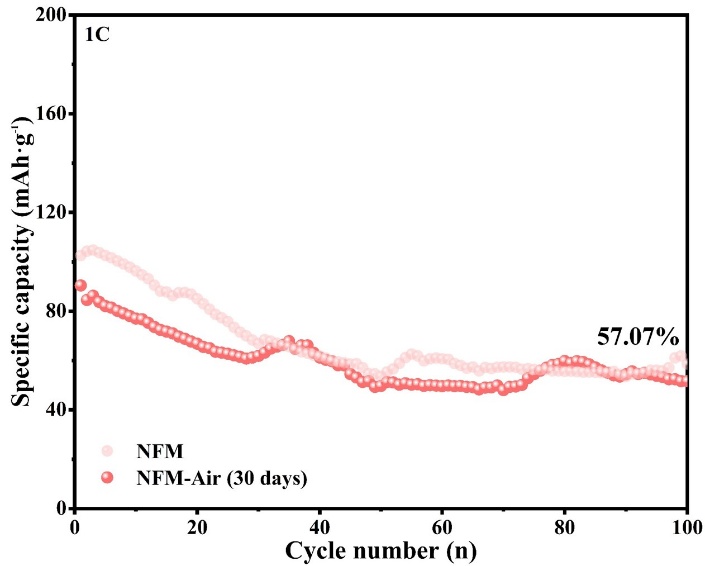
**

**Figure S20.** Long-term cycle test of NFMO exposed to moist air at 1 C.

**
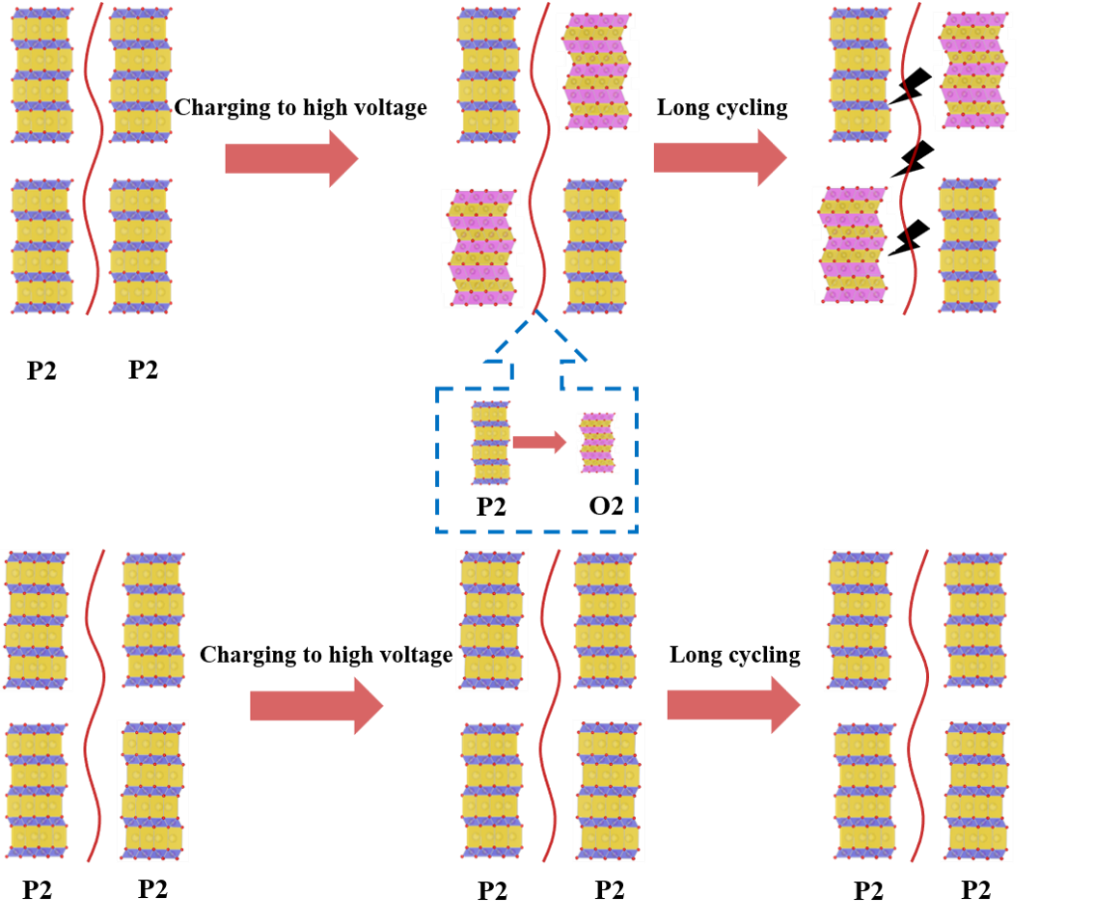
**

**Figure S21.** The phase change of NFMO and NFCMMOF during charge and discharge process.


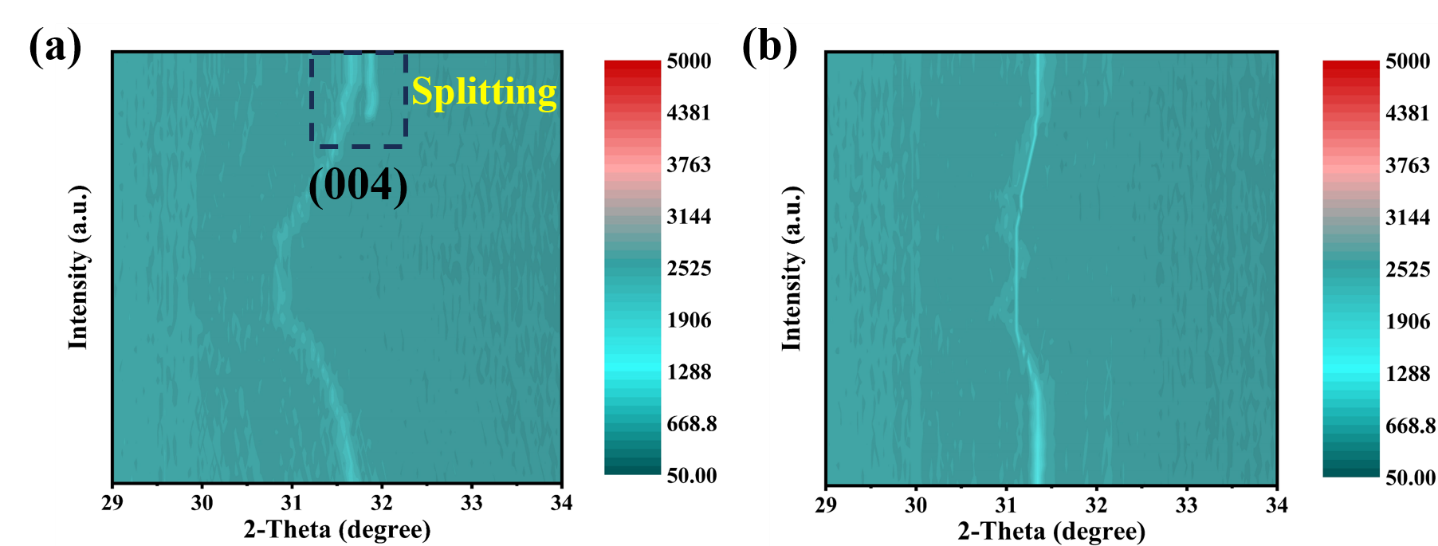


**Figure S22. (**004) diffraction peak enlarged image: (a) NFMO; (b) NFCMMOF.


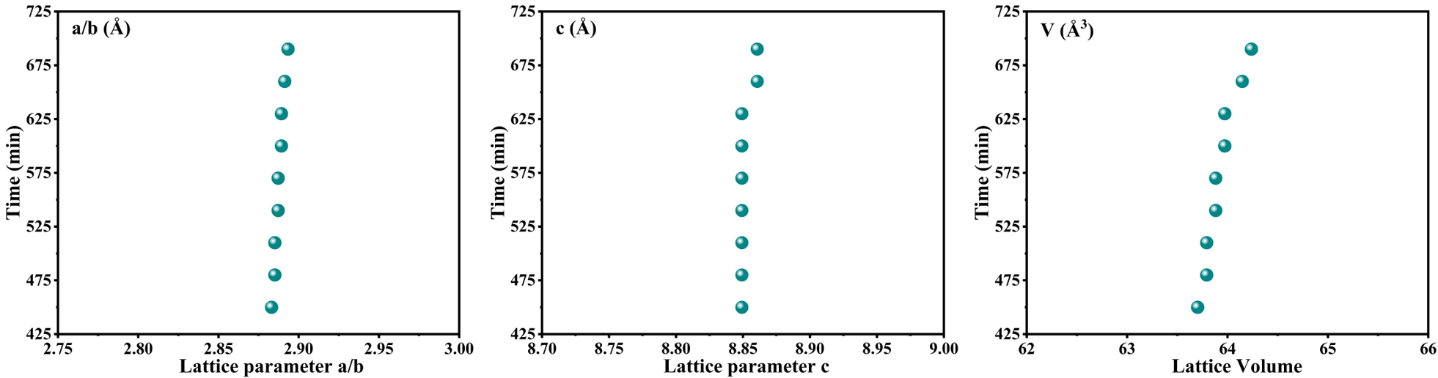


**Figure S23.** The change of O2 phase cell parameters of NFMO.

**
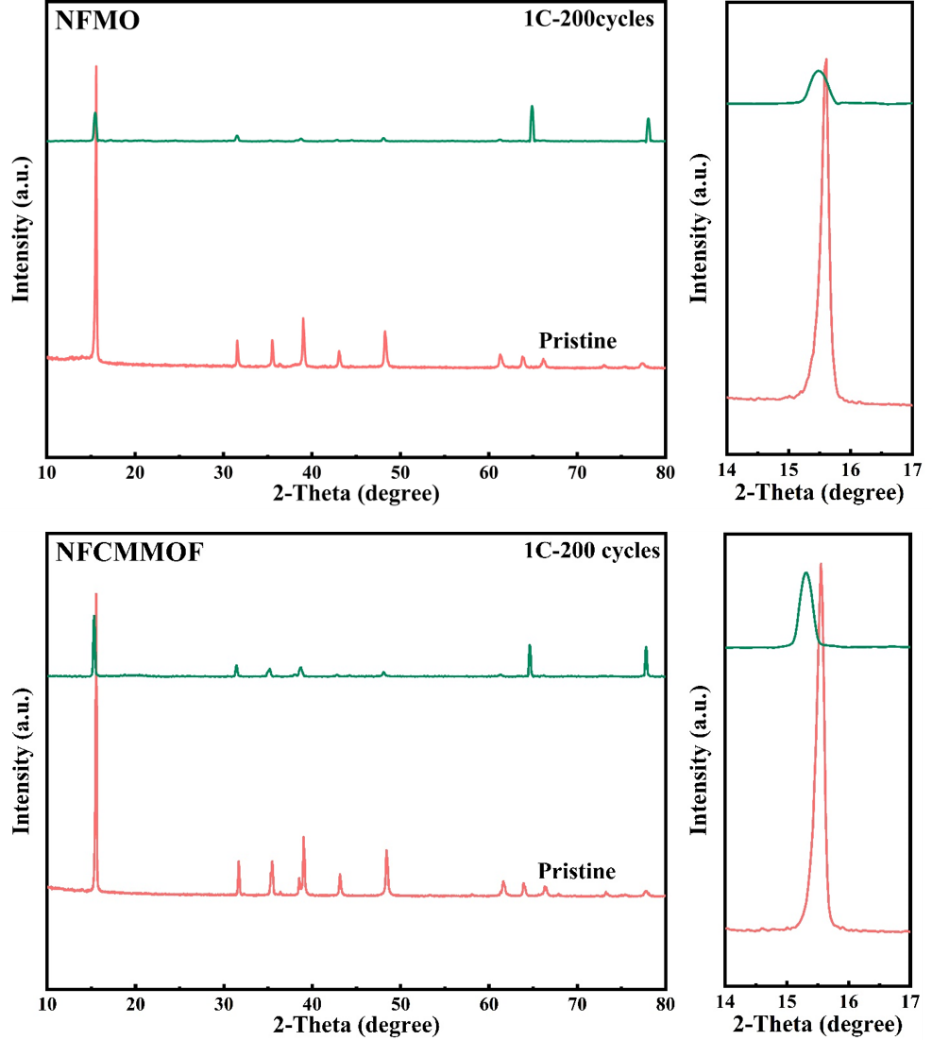
**

**Figure S24.** XRD test images of NFMO and NFCMMOF after 200 cycles at 1 C rate.


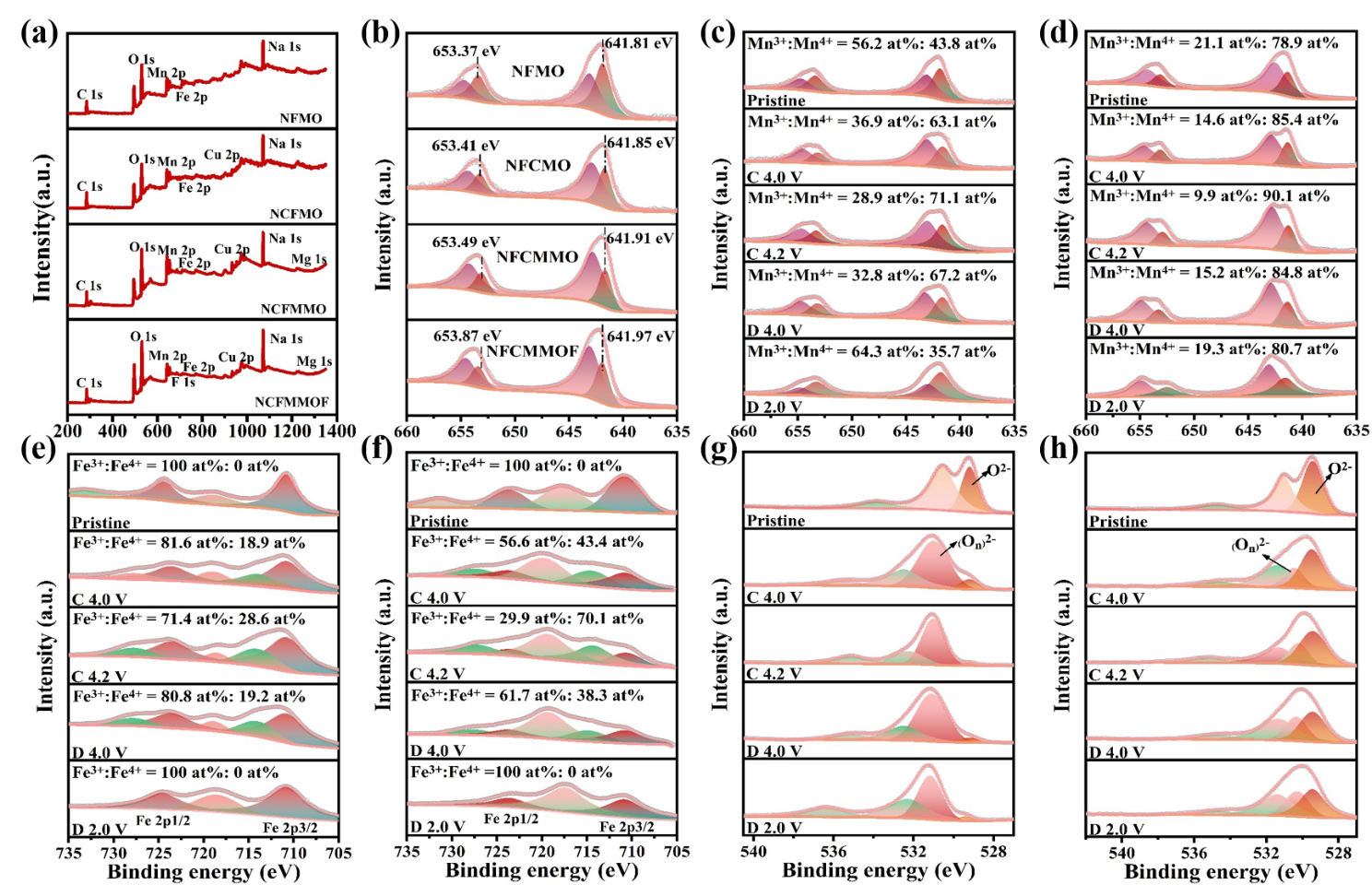


**Figure S25. Analysis of XPS valence change during charge and discharge process.** (a) Comparison of XPS spectra of NFMO, NFCMO, NFCMMO and NFCMMOF. (b) Comparison of Mn 2p spectra of NFMO, NFCMO, NFCMMO and NFCMMOF. The valence state of Mn changes in the process of charging and discharging: (c) NFMO; (d) NFCMMOF. The valence state of Fe changes in the process of charging and discharging: (e) NFMO; (f) NFCMMOF. The valence state of O changes in the process of charging and discharging: (g) NFMO; (h) NFCMMOF.

**
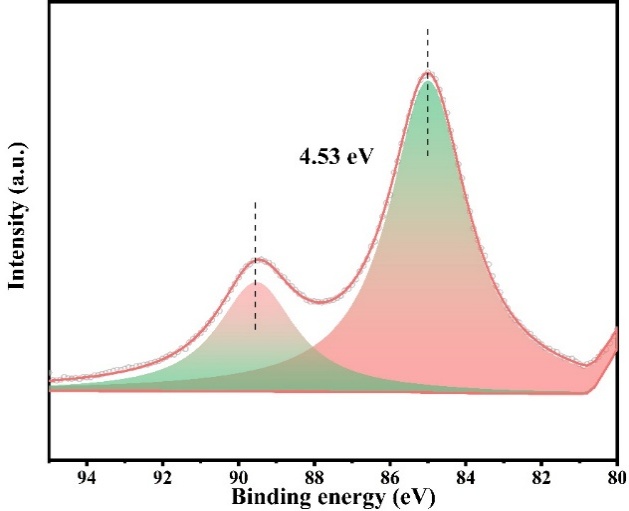
**

**Figure S26.** Spectra of Mn 3s in NFCMMOF.

**
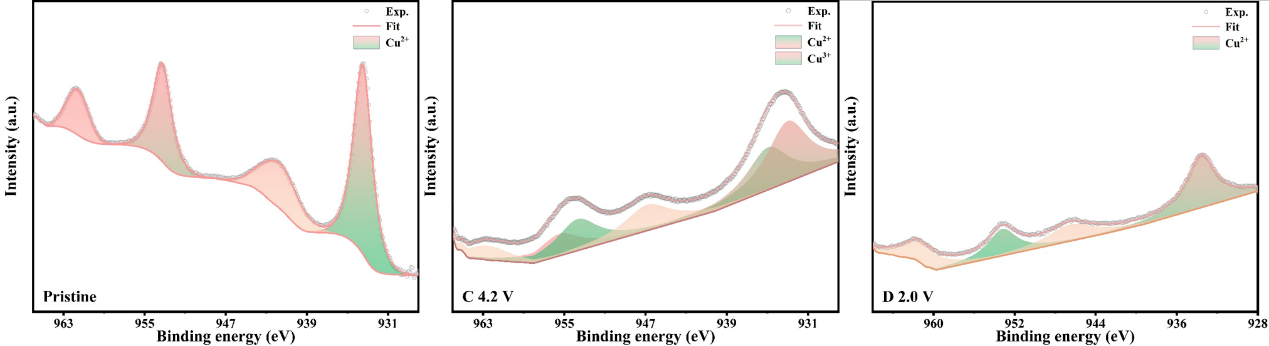
**

**Figure S27.** The change of valence state of Cu 2p in NFCMMOF during charge-discharge process.


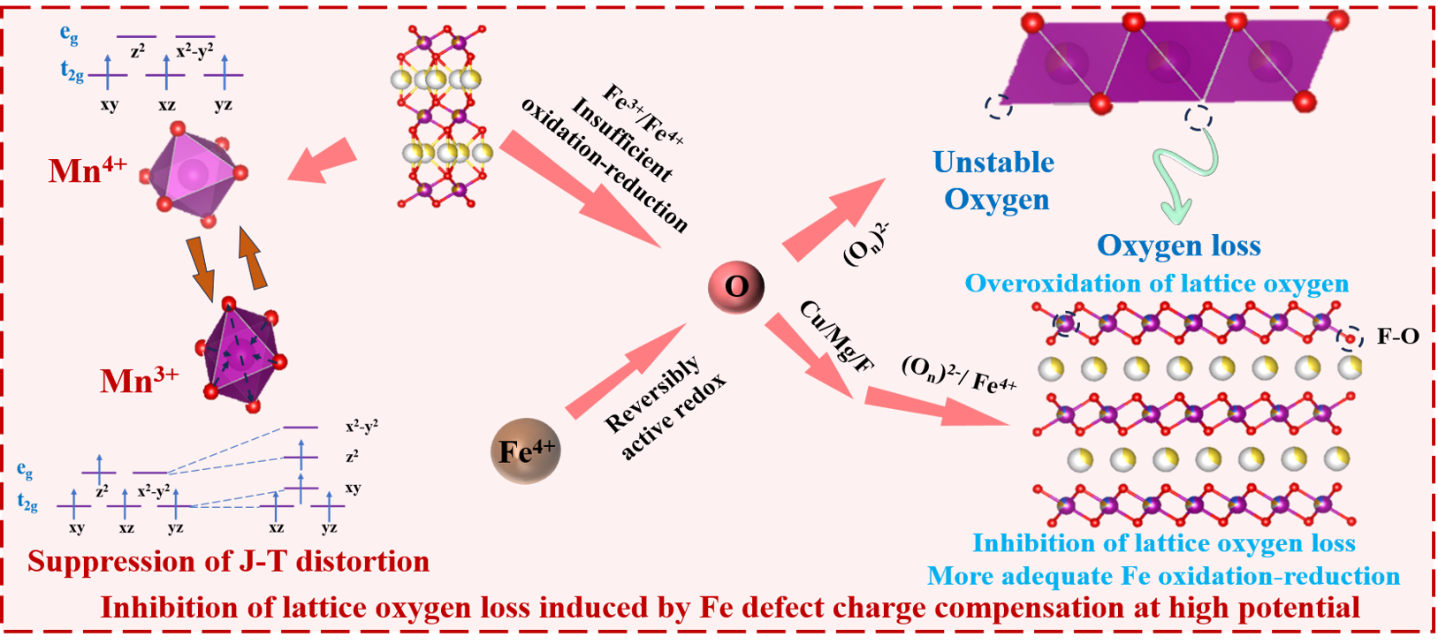


**Figure S28.** The inhibition of J-T distortion and the mechanism of lattice oxygen loss induced by charge compensation of high potential Fe defects.


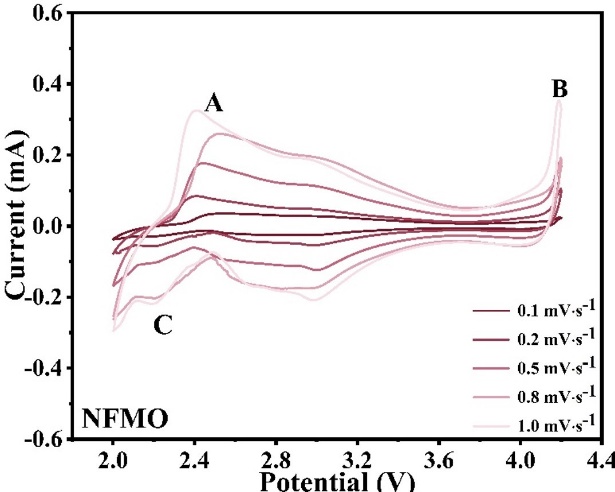


**Figure S29.** CV test of NFMO at different scan rates.


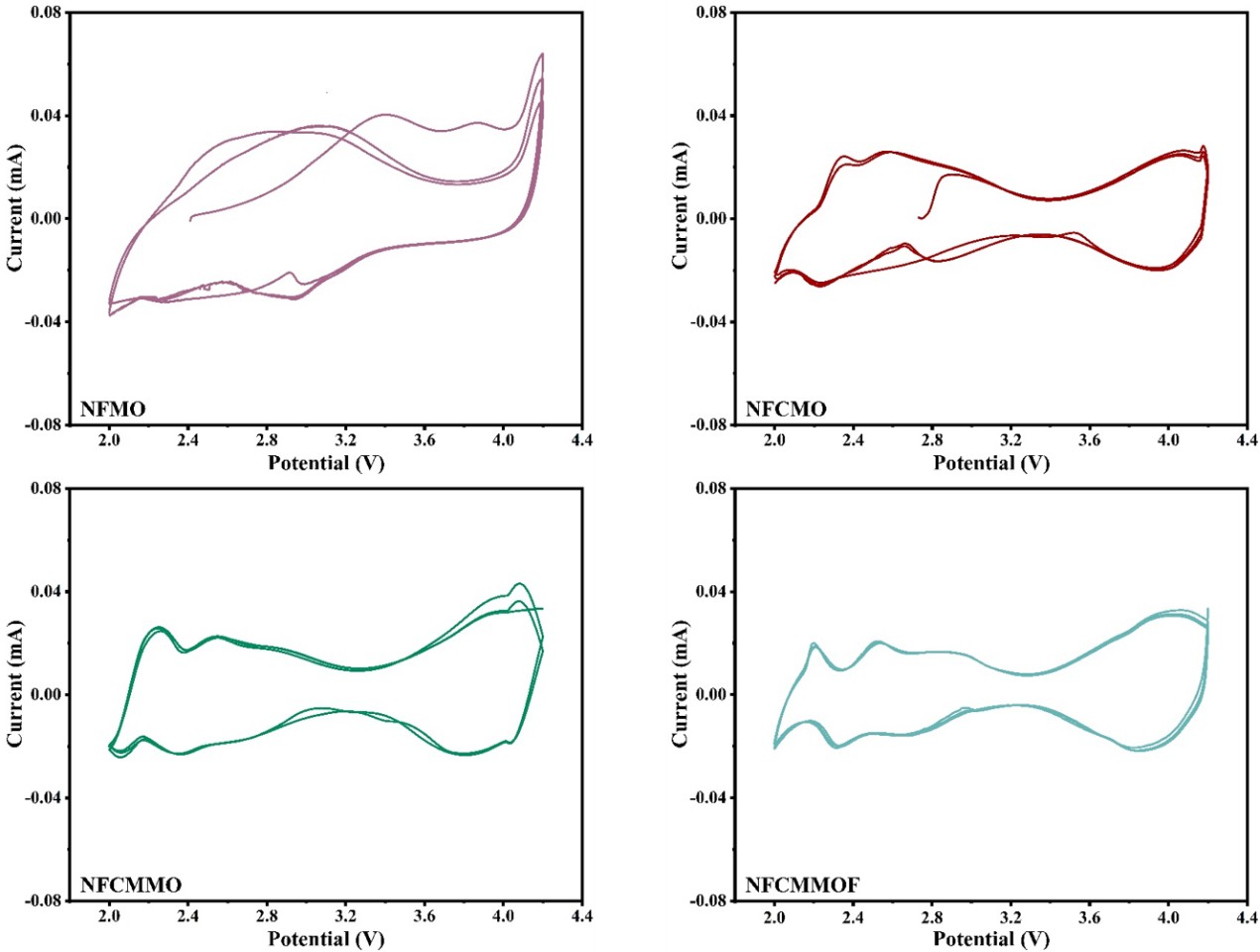


**Figure S30.** CV test at a scan rate of 0.1 mV/s.

**
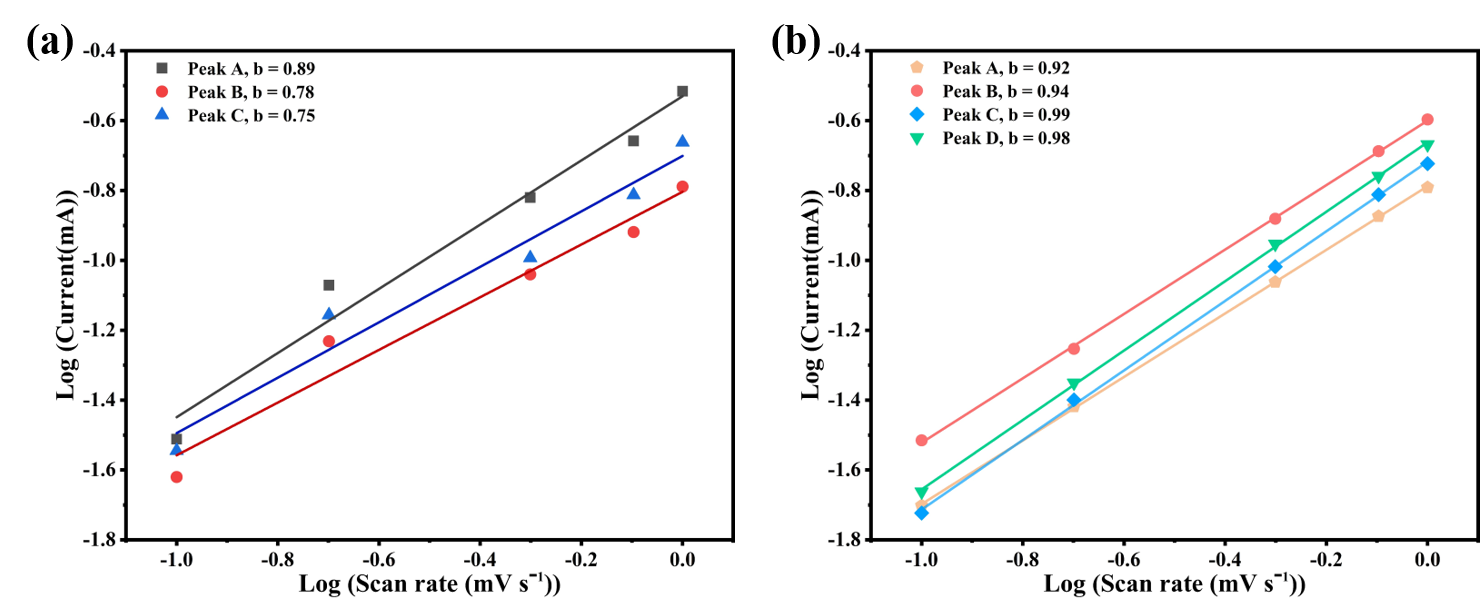
**

**Figure S31.** The function of the peak current of (a) NFMO and (b) NFCMMOF with the square root of the scan rate.

**
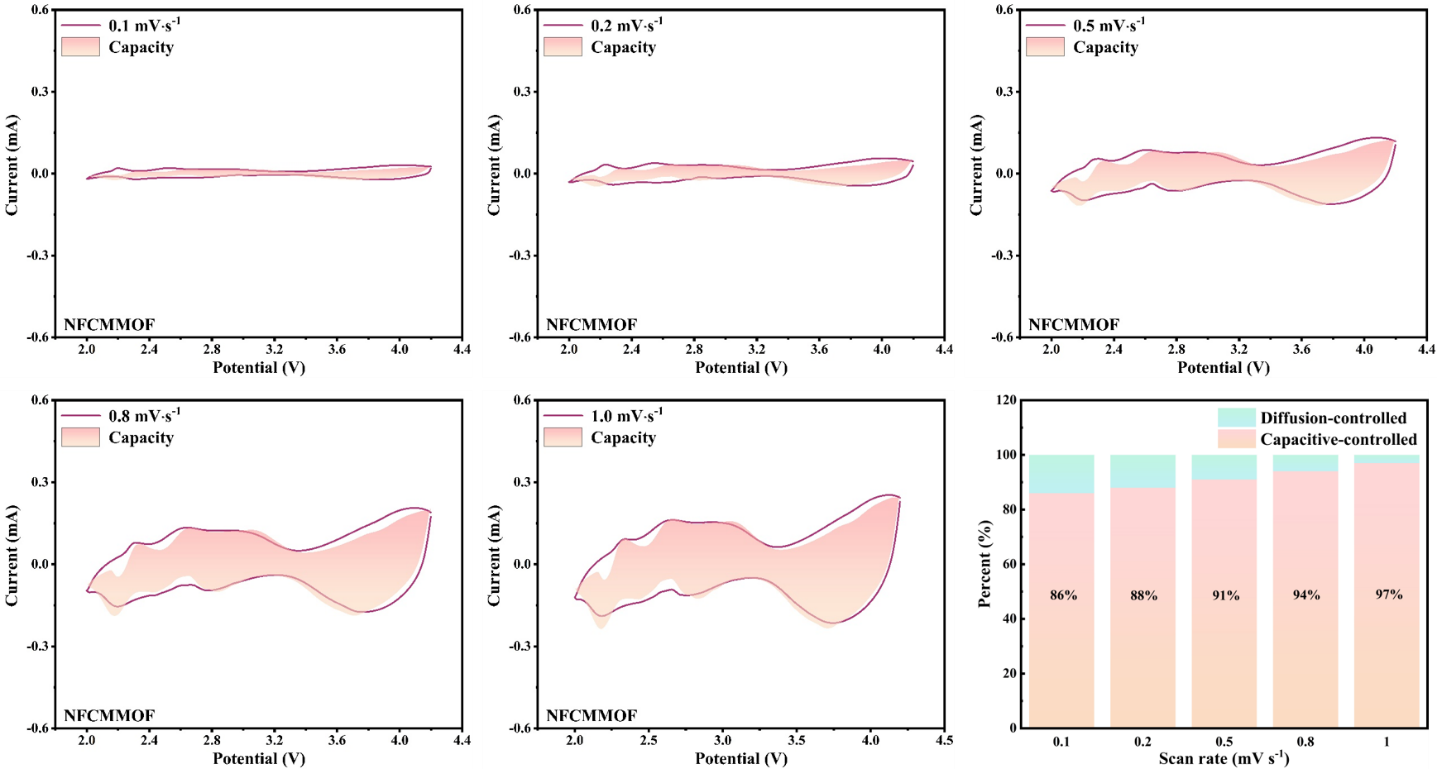
**

**Figure S32.** The pseudo-capacitance contribution of NFCMMOF at different scan rates.

**
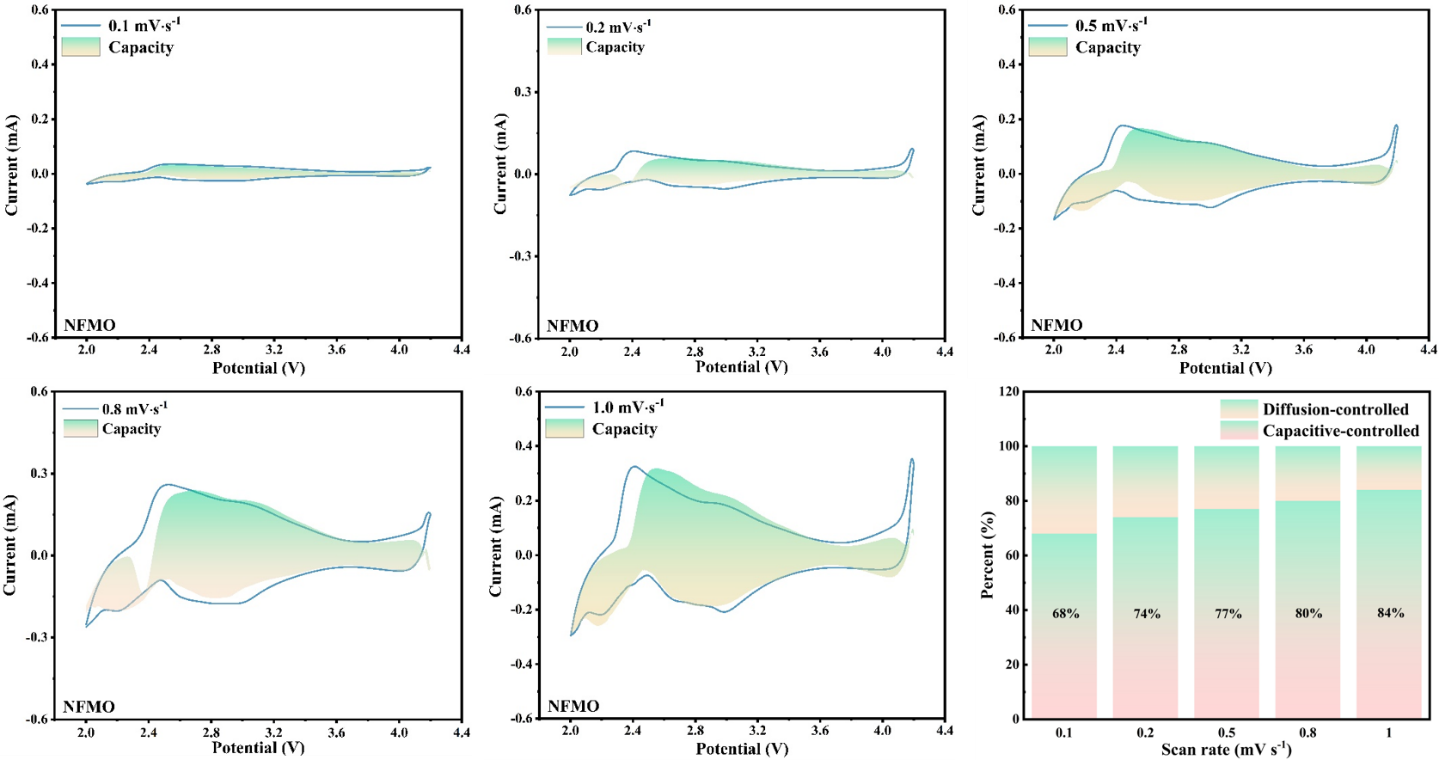
**

**Figure S33.** The pseudo-capacitance contribution of NFMO at different scan rates.


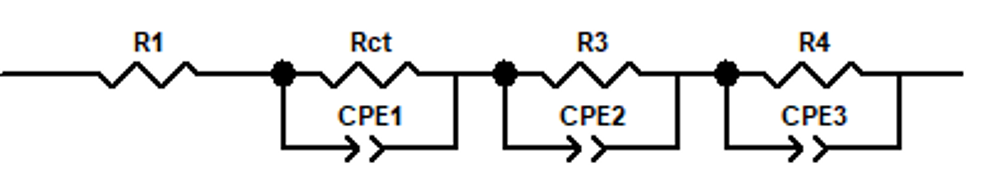


**Figure S34.** EIS fitting equivalent circuit diagram.


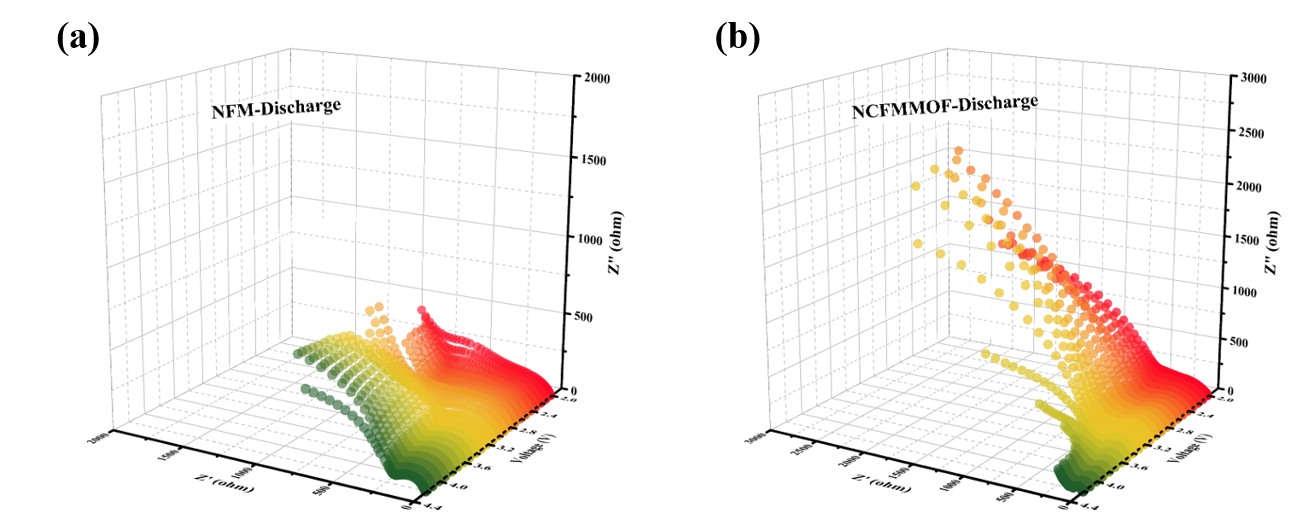


**Figure S35.** In-situ EIS three-dimensional image of discharge process: (a) NFMO; (b) NFCMMOF.


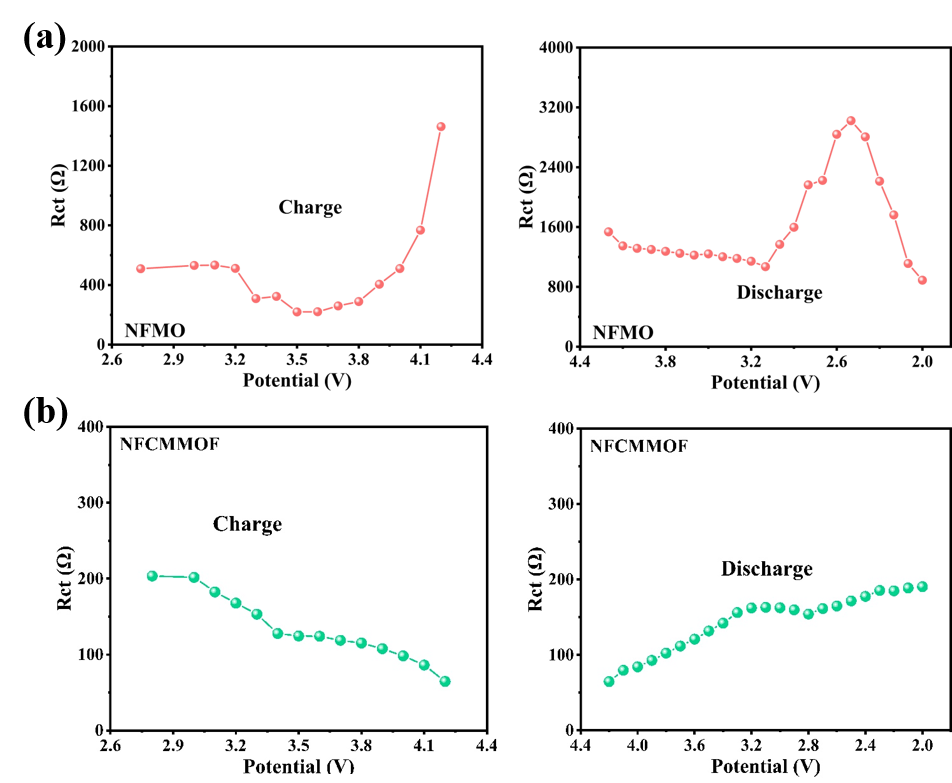


**Figure S36.** The R_ct_ value curve after in-situ EIS fitting: (a) NFMO; (b) NFCMMOF.


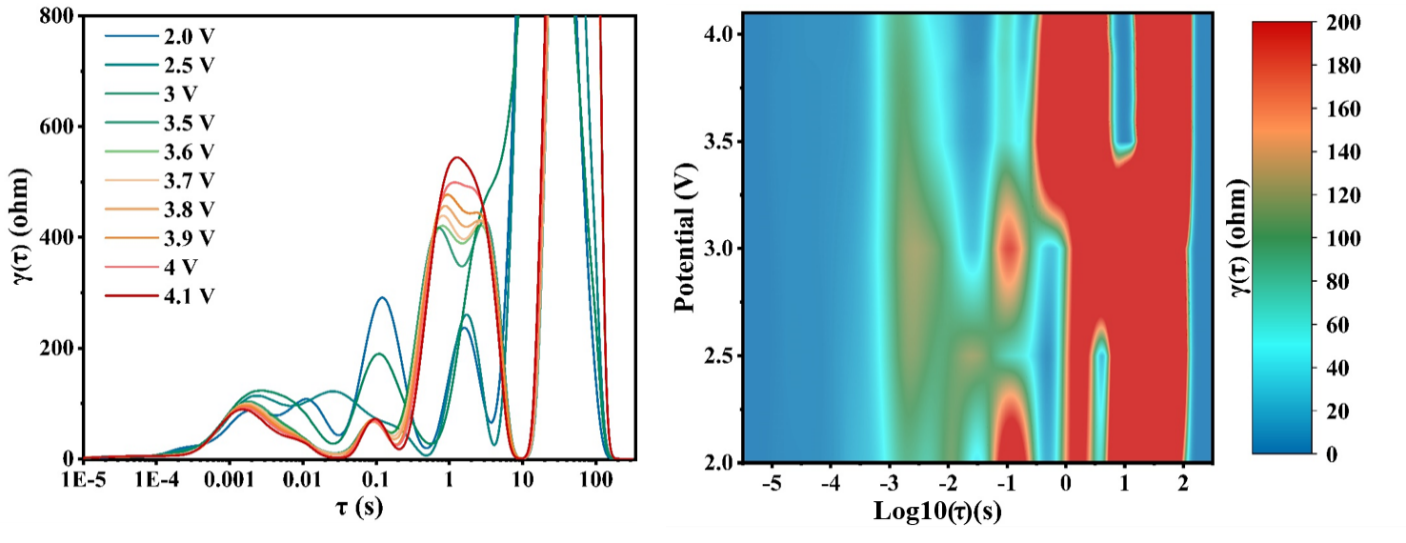


**Figure S37.** The corresponding relaxation time distribution (DRT) curve and contour map of NFMO were measured by potential-based in situ electrochemical impedance spectroscopy (In situ-EIS).


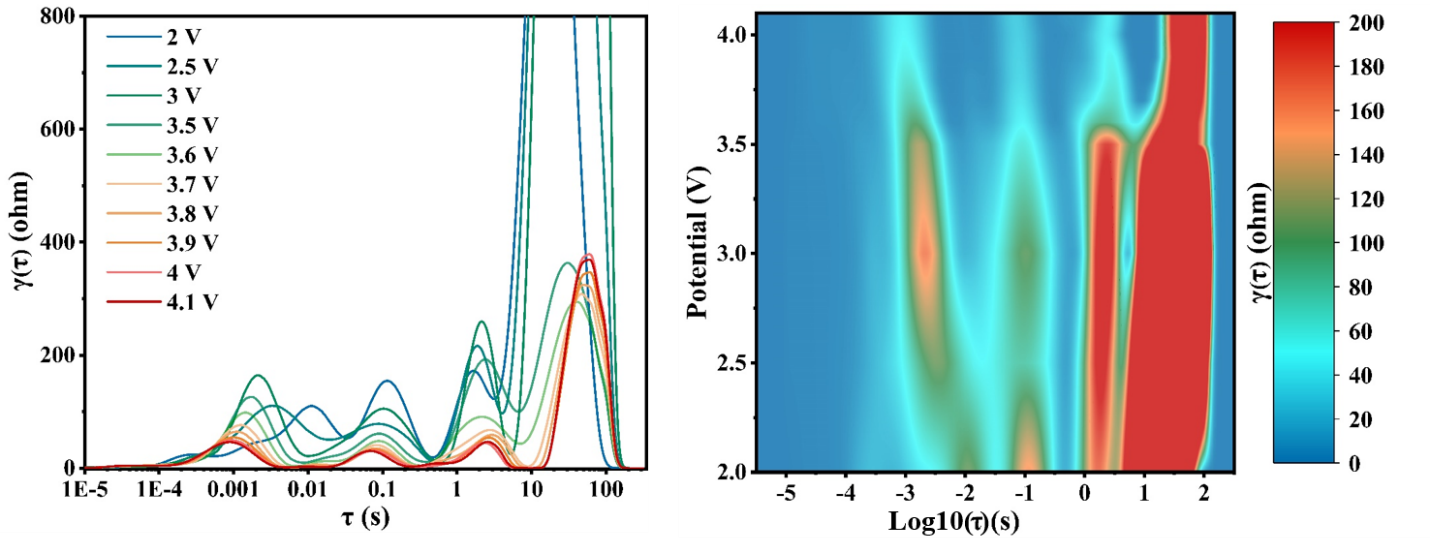


**Figure S38.** The corresponding relaxation time distribution (DRT) curve and contour map of NFCMMOF were measured by potential-based in situ electrochemical impedance spectroscopy (In situ-EIS).


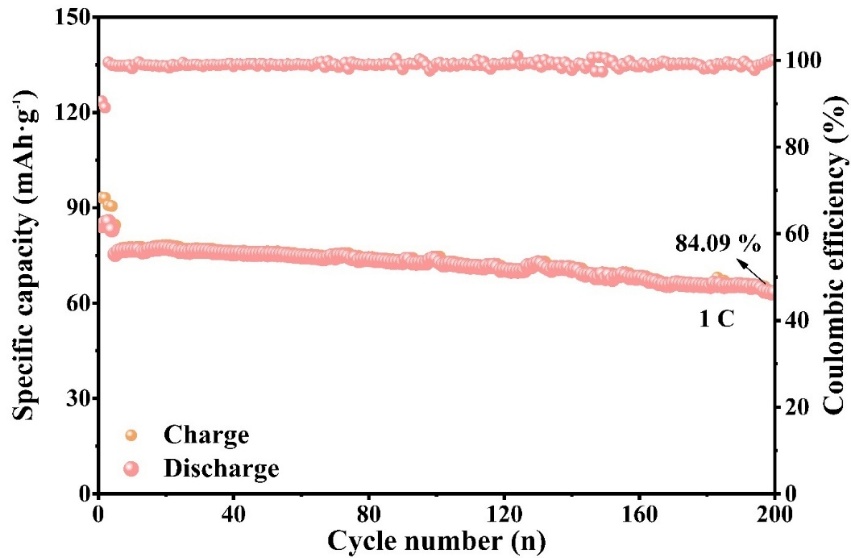


**Figure S39.** Long cycle test of NFCMMOF//HC full cell at 1 C.

**Table S1.** Detailed Rietveld refinement results for NFMO.

| Atom | Site | x | y | z | Occ. |
| --- | --- | --- | --- | --- | --- |
| Na1 | 2b | 0.00000 | 0.00000 | 0.25000 | 0.27 |
| Na2 | 2d | 0.66667 | 0.33333 | 0.25000 | 0.40 |
| Mn1 | 2a | 0.00000 | 0.00000 | 0.00000 | 0.67 |
| Fe1 | 2a | 0.00000 | 0.00000 | 0.00000 | 0.33 |
| O1 | 4f | 0.66667 | 0.33333 | 0.07541 | 1.00 |
| Space group: P_63/mmc_, a=b= 2.91572 Å, c= 11.31527 Å, α=β=90°, γ=120° | | | | | |
| R_wp_=8.6%, R_p_= 5.8%, Chi2: 2.16 | | | | | |

**Table S2.** Detailed Rietveld refinement results for NFCMO.

| Atom | Site | x | y | z | Occ. |
| --- | --- | --- | --- | --- | --- |
| Na1 | 2b | 0.00000 | 0.00000 | 0.25000 | 0.32 |
| Na2 | 2d | 0.66667 | 0.33333 | 0.25000 | 0.35 |
| Mn1 | 2a | 0.00000 | 0.00000 | 0.00000 | 0.67 |
| Fe1 | 2a | 0.00000 | 0.00000 | 0.00000 | 0.08 |
| Cu1 | 2a | 0.00000 | 0.00000 | 0.00000 | 0.25 |
| O1 | 4f | 0.66667 | 0.33333 | 0.09317 | 1.00 |
| Space group: P_63/mmc_, a=b= 2.90805 Å, c= 11.23083 Å, α=β=90°, γ=120° | | | | | |
| R_wp_= 8.7%, R_p_= 4.3%, Chi2: 2.34 | | | | | |

**Table S3.** Detailed Rietveld refinement results for NFCMMO.

| Atom | Site | x | y | z | Occ. |
| --- | --- | --- | --- | --- | --- |
| Na1 | 2b | 0.00000 | 0.00000 | 0.25000 | 0.30 |
| Na2 | 2d | 0.66667 | 0.33333 | 0.25000 | 0.37 |
| Mn1 | 2a | 0.00000 | 0.00000 | 0.00000 | 0.62 |
| Fe1 | 2a | 0.00000 | 0.00000 | 0.00000 | 0.08 |
| Cu1 | 2a | 0.00000 | 0.00000 | 0.00000 | 0.25 |
| Mg1 | 2a | 0.00000 | 0.00000 | 0.00000 | 0.05 |
| O1 | 4f | 0.66667 | 0.33333 | 0.08252 | 1.00 |
| Space group: P_63/mmc_, a=b= 2.91047 Å, c= 11.26722 Å, α=β=90°, γ=120° | | | | | |
| R_wp_= 8.2%, R_p_= 5.4%, Chi2: 2.17 | | | | | |

**Table S4.** Detailed Rietveld refinement results for NFCMMOF.

| Atom | Site | x | y | z | Occ. |
| --- | --- | --- | --- | --- | --- |
| Na1 | 2b | 0.00000 | 0.00000 | 0.25000 | 0.22 |
| Na2 | 2d | 0.66667 | 0.33333 | 0.25000 | 0.45 |
| Mn1 | 2a | 0.00000 | 0.00000 | 0.00000 | 0.62 |
| Fe1 | 2a | 0.00000 | 0.00000 | 0.00000 | 0.08 |
| Cu1 | 2a | 0.00000 | 0.00000 | 0.00000 | 0.25 |
| Mg1 | 2a | 0.00000 | 0.00000 | 0.00000 | 0.05 |
| F1 | 4f | 0.66667 | 0.33333 | 0.08133 | 0.025 |
| O1 | 4f | 0.66667 | 0.33333 | 0.08133 | 0.975 |
| Space group: P_63/mmc_, a=b= 2.91185 Å, c= 11.35210 Å, α=β=90°, γ=120° | | | | | |
| R_wp_= 7.4%, R_p_= 4.1%, Chi2: 2.03 | | | | | |

**Table S5.** Inductively coupled plasma optical emission spectrometry (ICP-OES) of NFMO

|  | **Na** | **Fe** | **Mn** |
| --- | --- | --- | --- |
| **NFMO** | 0.67 | 0.33 | 0.67 |

**Table S6.** Inductively coupled plasma optical emission spectrometry (ICP-OES) of NFCMO

|  | **Na** | **Fe** | **Mn** | **Cu** |
| --- | --- | --- | --- | --- |
| **NFCMO** | 0.67 | 0.08 | 0.67 | 0.25 |

**Table S7.** Inductively coupled plasma optical emission spectrometry (ICP-OES) of NFCMMO

|  | **Na** | **Fe** | **Mn** | **Cu** | **Mg** |
| --- | --- | --- | --- | --- | --- |
| **NFCMMO** | 0.67 | 0.08 | 0.62 | 0.25 | 0.05 |

**Table S8.** Inductively coupled plasma optical emission spectrometry (ICP-OES) of NFCMMOF

|  | **Na** | **Fe** | **Mn** | **Cu** | **Mg** | **F** |
| --- | --- | --- | --- | --- | --- | --- |
| **NFCMMOF** | 0.67 | 0.08 | 0.62 | 0.25 | 0.05 | 0.05 |

**Table S9.** Bader charge analysis was performed to evaluate the local charge distribution, where TM represents transition-metal sites, O1 corresponds to adjacent oxygen atoms, and O2 denotes the oxygen sites replaced by F.

|  | **TM** | **O1** | **O2** |
| --- | --- | --- | --- |
| **NFMO** | -1.1469 | 0.8245 | 0.8267 |
| **NFCMMOF** | -1.6142 | 0.9314 | 0.7218 |

**Table S10.** Comparison of electrochemical properties of different sodium ion cathode layered metal oxide materials

| **Material** | **Doping ions** | **Capacity**  **/mAh** **g^–1^** | **Capacity retention** |
| --- | --- | --- | --- |
| Na_0.65_Li_0.08_Cu_0.08_Fe_0.24_Mn_0.6_O_2_^[5]^ | Zn, Al | 129.5  (10 mA g^–1^) | 80.4%  (1400cycles, 1000 mA g^–1^) |
| Na_0.75_Zn_0.28_Mn_0.72_O_1.93_F_0.07_^[6]^ | F | 178.6  (18 mA g^–1^) | 83.1%  (400 cycles, 540 mA g^–1^) |
| Na_0.75_Mg_0.1_Ni_0.23_Mn_0.67_O_1.95_F_0.05_^[7]^ | Mg, F | 116  (75 mA g^–1^) | 73.0%  (1000 cycles, 1500 mA g^–1^) |
| P2-Na_0.67_[Cu_0.2_Co_0.2_Mn_0.6_O_2_^[8]^ | Cu/Co | 133  (10 mA g^–1^) | 93.0%  (100 cycles, 100 mA g^–1^) |
| Na_2/3_Ni_0.25_Mg_0.083_Mn_0.55_Ti_0.117_O_2_^[9]^ | Mg/Ti | 137  (17 mA g^–1^) | 77%  (200 cycles, 340 mA g^–1^) |
| Na_0.7_Cu_0.2_Fe_0.2_Mn_0.5_Ti_0.1_O_2_^[10]^ | Cu/Ti | 130  (10 mA g^–1^) | 71.1%  (300 cycles, 500 mA g^–1^) |
| K_0.05_Na_0.8_Ni_0.5_Mn_0.5_O_2_^[11]^ | K | 134.3  (20 mA g^–1^) | 83.17%  (200 cycles, 200 mA g^–1^) |
| Na_0.7_Li_0.03_Mg_0.03_Ni_0.27_Mn_0.6_Ti_0.07_O_2_^[12]^ | Li/Mg/Ti | 137  (17.3 mA g^–1^) | 82%  (200 cycles, 346 mA g^–1^) |
| **Na_0.67_Fe_0.08_Cu_0.25_ Mn_0.62_Mg_0.05_O_1.95_F_0.05_**  **(This work)** | Cu/Mg/F | 162.9  (20 mA g^–1^) | 85.14%  (1000 cycles, 2000 mA g^–1^)  91.14%  (500 cycles, 1000 mA g^–1^) |

**Table S11.** Comparison of electrochemical performance of different sodium ion cathode layered metal oxide materials for full battery.

| **Material** | **Current rate**  **/mA** **g^–1^** | **Capacity retention** |
| --- | --- | --- |
| Na_7/9_Cu_2/9_Fe_1/9_Mn_2/3_O_2_//Hard carbon^[13]^ | 20 | 79.00%  (50cycles) |
| Na_0.76_Cu_0.22_Fe_0.30_Mn_0.48_O_2_//hard carbon^[14]^ | 12 | 82.00%  (100 cycles) |
| Na_0.67_Ni_0.23_Mg_0.1_Mn_0.67_O_2_//HC^[15]^ | 170 | 75.50%  (100 cycles) |
| Na_0.67_Ni_0.33_Mn_0.67_O_2_-PMCs//HC^[16]^ | 170 | 69.60%  (100 cycles) |
| Na_0.67_Zn_0.05_Ni_0.15_Fe_0.20_Mn_0.60_O_1.95_F_0.05_^[17]^ | 100 | 73.00%  (150 cycles) |
| Na_0.8_Li_0.12_Ni_0.22_Mn_0.66_O_1.95_F_0.05_^[18]^ | 200 | 78.00%  (200 cycles) |
| **Na_0.67_Fe_0.08_Cu_0.25_ Mn_0.62_Mg_0.05_O_1.95_F_0.05_**  **(This work)** | 200 | 84.09%  (200 cycles) |
| **Na_0.67_Fe_0.08_Cu_0.25_ Mn_0.62_Mg_0.05_O_1.95_F_0.05_**  **(This work)** | 1000 | 79.70%  (400 cycles) |

**References**

[1] G. K. A, J. F. b, *Computational Materials Science* **1996**, 6, 15.

[2] J. P. Perdew, K. Burke, M. Ernzerhof, *Physical Review Letters* **1998**, 77, 3865.

[3] P. E. Blchl, *Physical review. B, Condensed matter* **1995**, 50, 17953.

[4] S. Grimme, *Journal of Computational Chemistry* **2010**, 27, 1787.

[5] J. Dai, J. Li, Y. Yao, Y. R. Wang, M. Ma, R. Bai, Y. Zhu, X. Rui, H. Wu, Y. Yu, *ACS Nano* **2025**, 19, 11197.

[6] X. Song, R. Liu, J. Jin, X. Zhao, Y. Wang, Q. Shen, Z. Sun, X. Qu, L. Jiao, Y. Liu, *Energy Storage Mater.* **2024**, 69, 10.

[7] X. Wang, Z. Yang, D. Chen, B. Lu, Q. Zhang, Y. Hou, Z. Wu, Z. Ye, T. Li, J. Lu, *Advanced Functional Materials* **2025**, 35.

[8] B. Ku, H. Ahn, S. Lee, J. Ahn, M. Choi, J. Kang, H. Park, J. Kim, A. Y. Kim, H. G. Jung, *Energy Storage Mater.* **2023**, 62, 12.

[9] Y. Huang, Z. Yan, W. Luo, Z. Hu, Y. Huang, *Energy Storage Mater.* **2020**, 29.

[10] M. Yan, K. Xu, Y. X. Chang, Z. Xie, M. Yang, S. Xu, *Journal of Colloid and Interface Science* **2023**.

[11] Y. Duan, Z. H. Ma, Y. Y. Huang, S. Bao, J. L. Lu, *Journal of Colloid and Interface Science* **2025**, 682, 715.

[12] Z. Cheng, B. Zhao, Y.-J. Guo, L. Yu, B. Yuan, W. Hua, Y.-X. Yin, S. Xu, B. Xiao, X. Han, P.-F. Wang, Y.-G. Guo, *Advanced Energy Materials* **2022**, 12, 2103461.

[13] Y. Li, Z. Yang, S. Xu, L. Mu, L. Gu, Y. S. Hu, H. Li, L. Chen, *Adv. Sci.* **2015**, 2, 1500031.

[14] Q. Shen, X. Zhao, Y. Liu, Y. Li, J. Zhang, N. Zhang, C. Yang, J. Chen, *Adv. Sci.* **2020**, 7, 2002199.

[15] B. Peng, Z. Sun, L. Zhao, J. Li, G. Zhang, *Energy Storage Mater.* **2021**, 35, 620.

[16] B. Peng, Z. Sun, L. Zhao, S. Zeng, G. Zhang, *Batteries & Supercaps* **2021**, 4, 456.

[17] D. W. Chen, B. He, S. Jiang, X. L. Wang, J. Song, H. Chen, D. Xiao, Q. Zhao, Y. Meng, Y. J. Wang, *Chem. Eng. J.* **2025**, 510, 11.

[18] H. X. Ren, Q. N. Zhou, Y. Li, L. M. Zheng, Q. Ni, Q. J. Li, J. Qian, S. Q. Li, Y. Zhao, F. Wu, C. Wu, Y. Bai, *Energy Storage Mater.* **2025**, 76, 11.
